# Oxidized phospholipids regulate amino acid metabolism through MTHFD2 to facilitate nucleotide release in endothelial cells

**DOI:** 10.1038/s41467-018-04602-0

**Published:** 2018-06-12

**Authors:** Juliane Hitzel, Eunjee Lee, Yi Zhang, Sofia Iris Bibli, Xiaogang Li, Sven Zukunft, Beatrice Pflüger, Jiong Hu, Christoph Schürmann, Andrea Estefania Vasconez, James A. Oo, Adelheid Kratzer, Sandeep Kumar, Flávia Rezende, Ivana Josipovic, Dominique Thomas, Hector Giral, Yannick Schreiber, Gerd Geisslinger, Christian Fork, Xia Yang, Fragiska Sigala, Casey E. Romanoski, Jens Kroll, Hanjoong Jo, Ulf Landmesser, Aldons J. Lusis, Dmitry Namgaladze, Ingrid Fleming, Matthias S. Leisegang, Jun Zhu, Ralf P. Brandes

**Affiliations:** 1 Institute for Cardiovascular Physiology, Goethe University, Frankfurt am Main, 60590 Germany; 2 German Center for Cardiovascular Research (DZHK) (Partner site Rhine-Main), Frankfurt am Main, 60590 Germany; 3 Icahn Institute of Genomics and Multiscale Biology, Mount Sinai Icahn School of Medicine, New York, 10029 NY USA; 4 Sema4 Genomics (a Mount Sinai venture), Stamford, 06902 CT USA; 5 Department of Mathematics, Hebei University of Science and Technology, Shijiazhuang, 050018 Hebei China; 6 Institute for Vascular Signalling, Centre for Molecular Medicine, Goethe University, Frankfurt am Main, 60590 Germany; 7 Department of Vascular Biology and Tumor Angiogenesis, European Center for Angioscience (ECAS), Medical Faculty Mannheim, Heidelberg University, Mannheim, 68167 Germany; 8 Department of Cardiology, Charité-Universitätsmedizin Berlin, Campus Benjamin Franklin, Berlin, 12203 Germany; 9 German Center for Cardiovascular Research (DZHK) (Partner site Berlin), Berlin, 13316 Germany; 10 Wallace H. Coulter Department of Biomedical Engineering, Georgia Institute of Technology and Emory University, Atlanta, 30332 GA USA; 11 Institute of Clinical Pharmacology, Pharmazentrum Frankfurt/ZAFES, Faculty of Medicine, Goethe University, Frankfurt am Main, 60590 Germany; 12 Fraunhofer Institute of Molecular Biology and Applied Ecology—Project Group Translational Medicine and Pharmacology (IME-TMP), Frankfurt am Main, 60596 Germany; 13 Department of Integrative Biology and Physiology, University of California, Los Angeles, 90095 CA USA; 14 1st Department of Propaedeutic Surgery, University of Athens Medical School, Hippocration Hospital, Athens, 11364 Greece; 15 Department of Cellular and Molecular Medicine, University of Arizona, Tucson, 85724 AZ USA; 16 Berlin Institute of Health (BIH), Berlin, 10178 Germany; 17 Departments of Medicine, Microbiology and Human Genetics, University of California, Los Angeles, 90095 CA USA; 18 Institute of Biochemistry I, Goethe University, Frankfurt am Main, 60590 Germany

## Abstract

Oxidized phospholipids (oxPAPC) induce endothelial dysfunction and atherosclerosis. Here we show that oxPAPC induce a gene network regulating serine-glycine metabolism with the mitochondrial methylenetetrahydrofolate dehydrogenase/cyclohydrolase (MTHFD2) as a causal regulator using integrative network modeling and Bayesian network analysis in human aortic endothelial cells. The cluster is activated in human plaque material and by atherogenic lipoproteins isolated from plasma of patients with coronary artery disease (CAD). Single nucleotide polymorphisms (SNPs) within the MTHFD2-controlled cluster associate with CAD. The MTHFD2-controlled cluster redirects metabolism to glycine synthesis to replenish purine nucleotides. Since endothelial cells secrete purines in response to oxPAPC, the MTHFD2-controlled response maintains endothelial ATP. Accordingly, MTHFD2-dependent glycine synthesis is a prerequisite for angiogenesis. Thus, we propose that endothelial cells undergo MTHFD2-mediated reprogramming toward serine-glycine and mitochondrial one-carbon metabolism to compensate for the loss of ATP in response to oxPAPC during atherosclerosis.

## Abstract (web summary)

During atherosclerosis, endothelial cells release purines in response to oxidized phospholipids. Here, Hitzel et al. show that oxidized phospholipids activate an MTHFD2-regulated gene network in endothelial cells which reprograms amino acid metabolism towards production of purines and thus compensates for their loss.

## Introduction

The endothelium maintains the vascular homeostasis and limits atherosclerosis development^1^. Activation of the endothelium leads to a vicious cycle of reactive oxygen species (ROS) formation, inflammation and recruitment and activation of monocytes. ROS generated by inflammatory cells and the endothelium promote the oxidation of lipids contained in lipoproteins that further promotes endothelial activation^2^. The process of lipid oxidation has been well studied for polyunsaturated fatty acid side chains of phospholipids in membranes and lipoproteins. Oxidized 1-palmitoyl-2-arachidonoyl-sn-glycero-3-phosphocholine (oxPAPC) is a mixture of oxidized phospholipids commonly found in oxidized low density lipoproteins (oxLDL). OxPAPC accumulates in atherosclerotic lesions and at sites of chronic inflammation^3^.

OxPAPC disturbs endothelial cells by activating both pro- and anti-inflammatory pathways^4^. Exposure to oxPAPC alters the endothelial transcriptome^5^ and the complex changes are a consequence of various regulatory circuits acting in concert. If analyzed at a single time point, the endothelial responses to oxPAPC are overwhelmingly complex. To address this, we went beyond traditional transcriptome analyses of the endothelial response to oxPAPC and applied a systems level network approach.

Network models have been used for the identification of genes and gene clusters causal to cardiovascular pathology^6^, yet a comprehensive network model for endothelial cells is lacking. Here, we constructed molecular causal network models, based on a Bayesian method^7^, for human aortic endothelial cells (HAEC). Compared to other methods, Bayesian networks have several advantages for modeling complex biological processes. They model a causal predictive component for regulatory relationships as well as reveal key molecular drivers and they provide flexible platforms to incorporate various -omics data as prior knowledge^8^.

Here, we apply these techniques to study endothelial cell metabolic reprogramming in response to oxPAPC. We propose that oxPAPC targets amino acid metabolism governed by MTHFD2 to replenish endothelial purine pools. Based on this, a key role of MTHFD2 in angiogenesis as well as human atherosclerosis and CAD development could be inferred.

## Results

### Differential connectivity clusters in response to oxPAPC

**Fig. 1:**
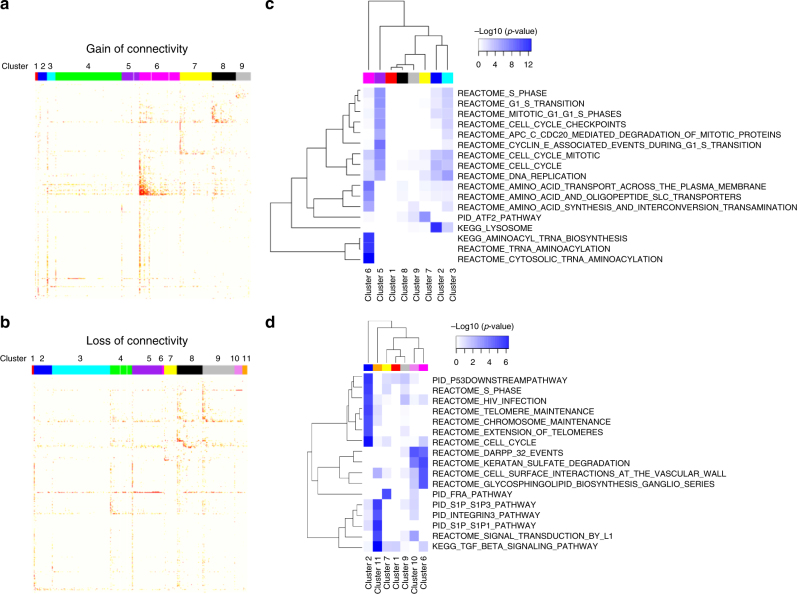
Differential connectivity clusters of HAEC reveal the emergence of novel gene clusters in response to oxidized phospholipids. **a**, **b** Topological overlap matrix of nine clusters with significant gain of connectivity (**a**) and 11 clusters with significant loss of connectivity (**b**) identified in a comparison of genome-wide gene−gene co-expression relationships between oxPAPC treated and control HAEC. **c**, **d** Heatmap of significantly overrepresented canonical pathways of gain of connectivity clusters (**c**) without cluster 4 (Supplementary Table 1) and loss of connectivity clusters (**d**) without clusters 3–5 and 8 (Supplementary Table 2) according to Fisher’s exact test are shown

To determine gene clusters and key drivers that shape the response of endothelial cells to pro-atherogenic lipids, an integrative network approach was applied (Supplementary Fig. 1). In the first step, expression profiles of HAEC obtained from 147 heart transplant donors were reanalyzed. In the initial experiments by Romanoski et al., HAECs of this cohort were exposed to oxPAPC (40 µg ml^−^^1^) and vehicle control for 4 h^5^. Rather than focusing on canonical analysis of differentially expressed genes or co-expression modules we went further and identified differentially connected gene pairs^9^ under the two conditions. This differential co-expression analysis is more sensitive to identify disease- or treatment-induced deregulation among interacting genes^9^. In total, 26,759 differentially connected gene pairs were significantly differentially co-expressed, among them 50.4% showed gain of connectivity (GOC), meaning enhanced co-regulation between genes with oxPAPC treatment. In all, 49.5% showed loss of connectivity (LOS). Clustered differentially connected gene pairs yielded nine significant GOS clusters (Fig. 1a) and 11 significant LOS clusters (Fig. 1b) with co-regulations elicited by oxPAPC. Gain of connectivity cluster 6 showed the most coherent differential connectivity changes compared to all other clusters. Thus, cluster 6 contains and reflects the strongest endothelial responses to oxPAPC. To identify differentially regulated biological processes, we compared each cluster with canonical pathways for enrichment (Fig. 1c, d, Supplementary Tables 1, 2). Gain of connectivity cluster 6 was significantly enriched for genes involved in amino acid-related biological processes (Fisher’s exact test (FET) *p* value = 1.63E-09) (Supplementary Table 3), hereafter termed amino acid cluster. The applied differential connectivity clustering approach suggests that amino acid metabolism experiences a massive remodeling in response to oxidized phospholipids.

### MTHFD2 is a key driver for the HAEC Bayesian network

**Fig. 2:**
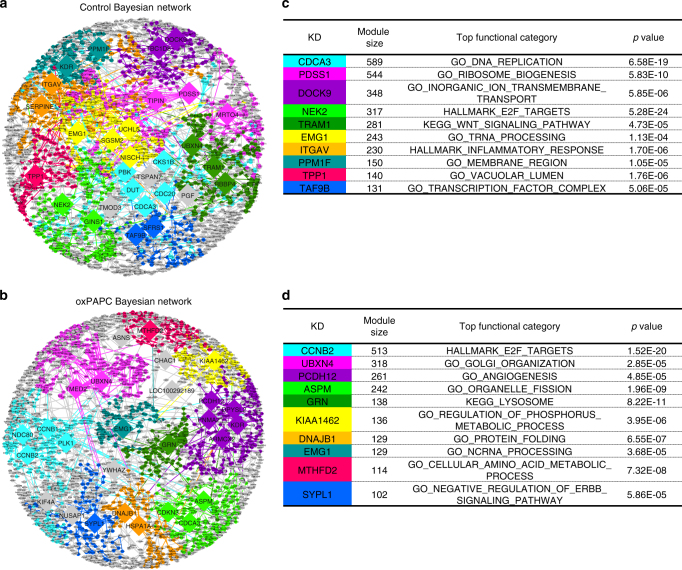
Bayesian networks of HAEC reveal novel key drivers in response to oxidized phospholipids. **a**, **b** Network view of HAEC Bayesian network of control state (**a**) and oxPAPC-treated state (**b**). Key drivers with more than 100 downstream nodes are indicated and ten top-ranked subnetworks according to strength of enrichment and subnetwork size are colored. Edges are colored according to source node. **c**, **d** List of ten subnetworks colored within control (**c**) and oxPAPC (**d**) Bayesian network with most top key driver, number of nodes, most significant overrepresented functional category and *p* value are listed

To explore the mechanisms underlying deregulated amino acid metabolism in oxPAPC-treated endothelial cells, we constructed molecular Bayesian networks. This approach allows for the identification of corresponding causal regulators. For network construction, we integrated the above used expression profiles and previously published expression quantitative trait loci^10^ of the same cohort. This allowed us to construct two Bayesian networks for HAEC: one for the control situation without oxPAPC (Fig. 2a, Supplementary Data 1) and one for oxPAPC exposure (Fig. 2b, Supplementary Data 2). Assessment of the control and oxPAPC Bayesian network against widely used databases showed that the estimated accuracy of these Bayesian networks is significantly higher than that of random networks (Supplementary Fig. 2a). Also, the proposed networks showed significantly better prediction of gene−gene interactions in endothelial cells as compared to global gene networks from the Human Protein Reference Database (Supplementary Fig. 2b, c). To identify causal regulators and the biological processes targeted by oxPAPC, we identified key drivers^7,11^ in the two networks and analyzed their associated subnetworks for enriched canonical pathways (Fig. 2c, d). The identified top ranked key drivers and their associated subnetworks (>100 nodes in subnetwork) are listed in the supplement (Supplementary Tables 4,5). *MTHFD2* was identified as a key driver specific for the oxPAPC Bayesian network (Fig. 2d). The subnetwork regulated by *MTHFD2*, termed *MTHFD2* network, was significantly enriched for genes involved in amino acid metabolism (FET *p* value = 7.32E-08) (Supplementary Table 6) and significantly overlapped with the identified amino acid cluster (FET *p* value = 2.33E-28). Thus, MTHFD2 may facilitate a shift in amino acid metabolism in HAEC in response to oxidized phospholipids.

### *MTHFD2-*associated amino acid metabolic gene cluster

**Fig. 3:**
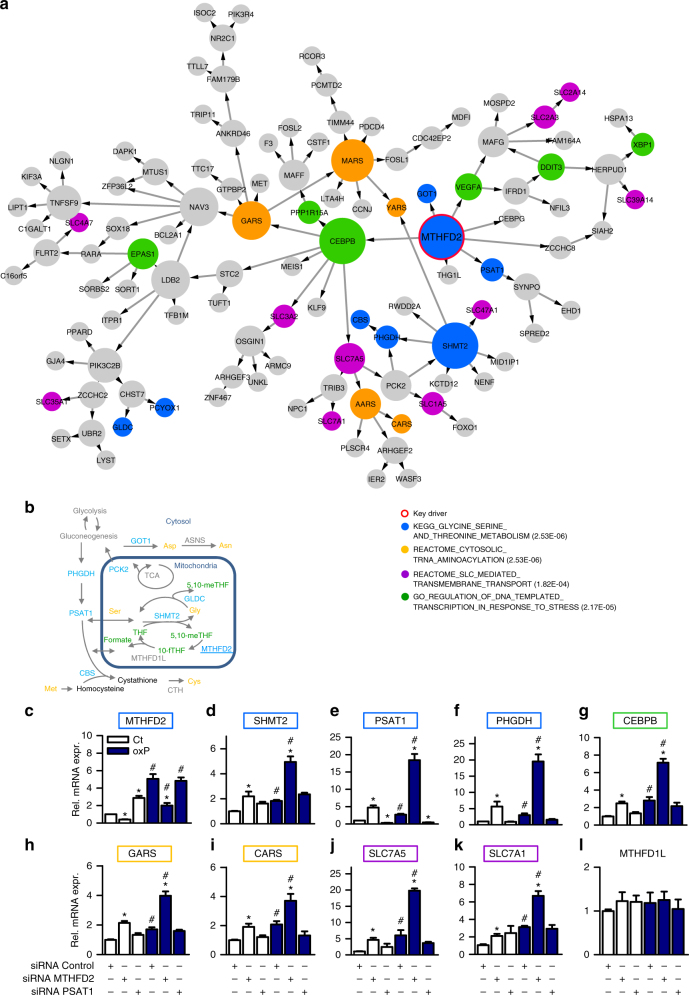
Key driver *MTHFD2* and oxPAPC induce endothelial Bayesian amino acid subnetwork. **a** Network view of the oxPAPC Bayesian subnetwork with *MTHFD2* as key driver. Nodes that belong to indicated significantly overrepresented canonical gene set categories are highlighted respectively. Node size reflects out-degree. **b** Schematic diagram of enzymes within the *MTHFD2* network (blue) that are directly or indirectly involved in serine, glycine, cysteine, methionine, aspartate, and asparagine (orange) interconversion as well as interconversion of tetrahydrofolates (THF) inside mitochondria (green). CBS: Cystathionine-Beta-Synthase, PCK2: Phosphoenolpyruvate Carboxykinase 2, GOT1 Glutamic-Oxaloacetic Transaminase 1, GLDC: Glycine Decarboxylase, ASNS: Asparagine Synthetase. **c−l** Experimental validation of the *MTHFD2* network as assessed by quantitative RT-PCR. HAEC with and without knockdown of the key driver *MTHFD2* or the downstream node *PSAT1* were exposed to medium (1% FCS) with (oxP) or without (Ct) oxPAPC for 4 h (*n* ≥ 4). Genes belonging to the *MTHFD2* network are framed by the color of the corresponding gene set category as in **a**. Data are represented as mean ± SEM, *p ≤ 0.05 (*MTHFD2* or *PSAT1* vs Control siRNA), ^#^*p* ≤ 0.05 (oxP vs Ct) (ANOVA with Bonferroni post-hoc test). MTHFD2: Methylenetetrahydrofolate Dehydrogenase (NADP + Dependent) 2-Methenyltetrahydrofolate Cyclohydrolase, SHMT2: serine hydroxymethyltransferase 2, PHGDH: phosphoglycerate dehydrogenase, PSAT1: phosphoserine aminotransferase 1, CEBPB: CCAAT/Enhancer Binding Protein Beta, GARS: Glycyl-TRNA Synthetase, CARS Cysteinyl-TRNA Synthetase, SLC7A5: Solute Carrier Family 7 Member 5, SLC7A1: Solute Carrier Family Member 1, MTHFD1L: Methylenetetrahydrofolate Dehydrogenase (NADP + Dependent) 1-Like

The endothelial *MTHFD2* network comprised 114 genes and was significantly enriched for cytosolic tRNA-aminoacylation (FET *p* value = 2.53E-06), membrane transport proteins of the solute carrier family (FET *p* value = 1.82E-04), the majority of which are involved in the import of amino acids, and transcriptional regulation in response to stress (FET *p* value = 2.17E-05) (Fig. 3a). Importantly, the *MTHFD2* network was also enriched for genes involved in glycine-serine metabolism (FET *p* value = 2.53E-06). The genes within the overlap of the *MTHFD2* network and this gene set category control mitochondrial folate cycle, glycine and serine metabolism and contribute to aspartate, asparagine, methionine, and cysteine interconversion (Fig. 3b).

MTHFD2 is highly induced in many cancers and is essential for mitochondrial one-carbon metabolism—the highest scoring metabolic pathway across human cancers^12^. MTHFD2 is a mitochondrial enzyme with 5,10-methylene-tetrahydrofolate (5,10-meTHF) dehydrogenase and cyclohydrolase activity. The mitochondrial folate cycle generates glycine from serine, which is catalyzed by the enzyme mitochondrial serine hydroxymethyltransferase 2 (*SHMT2*) (Fig. 3b). The serine consumed for this reaction is synthesized by the cytosolic phosphoglycerate dehydrogenase (*PHGDH*) and the phosphoserine aminotransferase 1 (*PSAT1*) from the glycolysis intermediate 3-phosphoglycerate^13^. During the reaction of SHMT2 the co-factor tetrahydrofolate (THF) is converted to 5,10-meTHF. The enzyme MTHFD2 uses 5,10-meTHF to produce the highly reactive 1-C donor 10-formylTHF (10-fTHF) which can be recycled to THF by MTHFD1L. Both 10-fTHF and glycine are indispensable for the de novo synthesis of purines^14^. Interestingly, the function of MTHFD2 in differentiated cells has been widely overlooked and its role in endothelial biology or in lipid signaling remains, to our knowledge, unknown. Given the important role of metabolism for endothelial cell reprogramming^15^, we further analyzed this interesting subnetwork.

### MTHFD2 mediated oxPAPC-dependent changes

To better understand and validate the *MTHFD2* network, we assessed the role of *MTHFD2* in endothelial cells. *MTHFD2* expression was decreased by siRNAs in HAEC and gene expression changes were determined with and without oxPAPC. To determine the hierarchical position of *MTHFD2* within the subnetwork, we additionally silenced *PSAT1*, one of its direct downstream nodes (Fig. 3a). Expression of *MTHFD2* was induced by oxPAPC as well as by knockdown of *PSAT1* (Fig. 3c). Importantly, also the expression of the key enzymes of the serine-glycine synthesis pathway (*SHMT2, PSAT1,* and *PHGDH*) was increased by oxPAPC, and this effect was potentiated by knockdown of *MTHFD2* (Fig. 3d–f). OxPAPC and *MTHFD2* knockdown, both and in an additive manner, increased the expression of the transcription factor *CEBPB* (Fig. 3g), the aminoacyl-tRNA synthetases for glycine (*GARS*) and cysteine (*CARS*) (Fig. 3h, i) and the solute carrier transporters *SLC7A5* and *SLC7A1* (Fig. 3j, k). Expression of genes in the *MTHFD2* network was similarly but to a lesser extent affected by *PSAT1* knockdown. Importantly, *MTHFD1L*, which contributes to mitochondrial one-carbon cycle, but does not belong to the *MTHFD2* network, was unaffected by oxPAPC and unaltered by siRNA against *PSAT1* or *MTHFD2* (Fig. 3l). These results validate *MTHFD2* as a key regulator of the *MTHFD2* network in oxPAPC-exposed HAEC. The data indicate that oxPAPC elicits a metabolic shift promoting the cellular uptake of amino acids and the de novo synthesis of glycine.

### *MTHFD2* regulates 18 genes related to amino acid metabolism

**Fig. 4:**
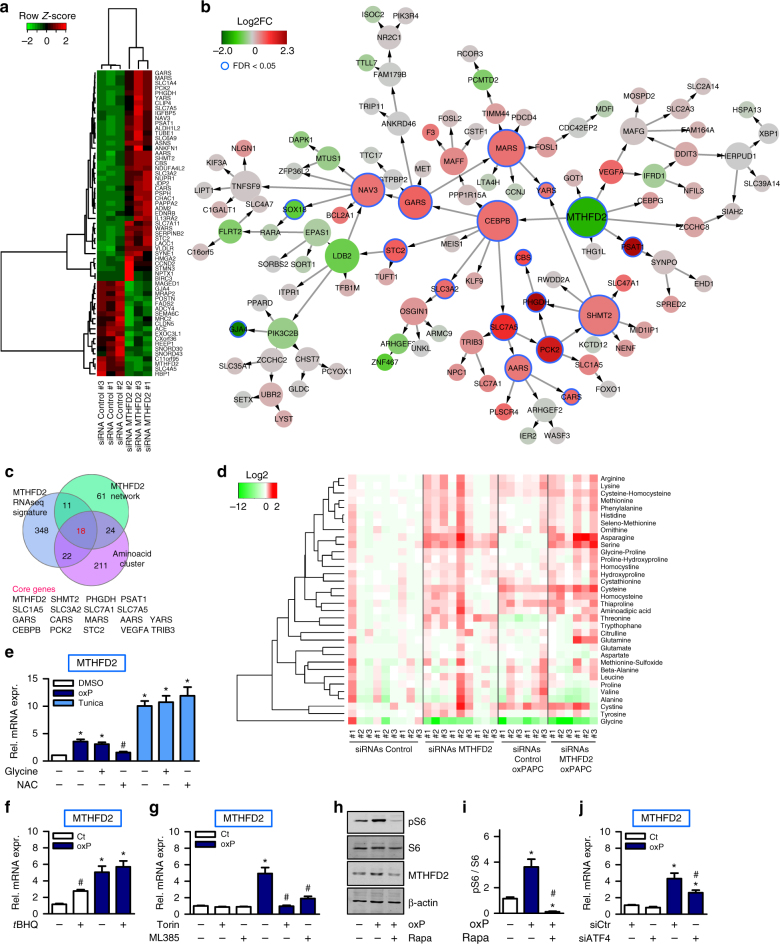
Knockdown of *MTHFD2* and oxPAPC drains the intracellular glycine pool. **a** Heatmap for fragments per kilobase of transcript per million mapped reads (FPKM) of significantly differentially expressed genes (FDR < 0.01, Benjamini−Hochberg). RNAseq was performed in HAEC with three different siRNAs against *MTHFD2* or scrambled control. **b** Projection of RNAseq signature (FDR < 0.01, Benjamini−Hochberg) in **a** onto the *MTHFD2* network. Direction of expression of nodes of the *MTHFD2* network in RNAseq signature is indicated by the node color. Node size reflects out-degree. **c** Venn diagram of genes in the amino acid cluster, *MTHFD2* network and *MTHFD2* RNAseq signature (FDR < 0.01, Benjamini−Hochberg). Genes belonging to all three gene sets are listed. **d** Heatmap of amino acid profile. HAEC were treated with three different siRNAs against *MTHFD2* or scrambled control and exposed to medium (1% FCS) with or without oxPAPC for 4 h. Amino acids in cell lysates were measured by mass spectrometry (*n* = 6–9). **e** Relative mRNA expression of *MTHFD2* in HAEC pretreated with *N*-acetylcysteine (NAC) (5 mM) and glycine (500 µM) for 1 h and then exposed to medium (1% FCS) with (oxP) or without (Ct) oxPAPC or tunicamycin (10 µg ml^−1^) for 4 h (*n* = 5). **f** qRT-PCR detection of MTHFD2 in HAEC exposed to medium (1% FCS) with or without oxPAPC and treated with *t*BHQ (20 µM) or DMSO as control for 24 h (*n* = 4). **g** HAEC were exposed to medium (1% FCS) with or without oxPAPC and additionally treated with torin (100 nM), ML385 (10 µM) or DMSO as control (*n* = 4). **h**, **i** Western blot detection (**h**) and quantification (**i**) of phosphorylated S6, S6 and MTHFD2 in HAEC pretreated with rapamycin (20 nM) or DMSO as control overnight and exposed to medium (1% FCS) with or without oxPAPC for 4 h (*n* = 4). **j** HAEC were treated with scrambled control siRNA (siCtr) or siRNA against *ATF4* and exposed to oxPAPC or control medium for 4 h (*n* = 5). Data are represented as mean ± SEM, *p ≤ 0.05 (oxP or tunicamycin vs Ct), ^#^*p* ≤ 0.05 (inhibitor present vs absent or *ATF4* vs Control siRNA) (ANOVA with Bonferroni post-hoc test)

To address the importance of *MTHFD2* for endothelial gene expression, we performed RNAseq of HAEC with and without siRNA against *MTHFD2* (Fig. 4a). Gene set enrichment analysis of the *MTHFD2* RNAseq signature confirmed a strong enrichment for amino acid metabolism (FET *p* value = 7.54E-14) (Supplementary Data 3). We then projected the *MTHFD2* RNAseq signature onto the *MTHFD2* network. This demonstrated that *MTHFD2*′s close neighbors were upregulated by *MTHFD2* siRNA (Fig. 4b). Furthermore, the *MTHFD2* RNAseq signature overlapped with the *MTHFD2* network as compared to the whole oxPAPC Bayesian network (FET *p* value = 8.34E-16). Finally, the *MTHFD2* RNAseq signature also significantly overlapped with the amino acid cluster as compared to all other differential connectivity clusters (FET *p* value = 1.19E-17).

To identify indispensable pathway genes, an integrative analysis of all three data sets, the amino acid cluster, the *MTHFD2* network and the *MTHFD2* RNAseq signature, was carried out. This yielded 18 commonly shared genes, which included key enzymes of the glycine-serine-synthesis pathway, aminoacyl-tRNA synthetases, solute carrier transporters as well as the transcription factor *CEBPB* and the mitochondrial phosphoenolpyruvate carboxykinase 2 (*PCK2*) (Fig. 4c). Collectively, these data further support the role of MTHFD2 in amino acid metabolic reprogramming.

### OxPAPC and *MTHFD2* siRNA deplete intracellular glycine pools

We next identified the physiological function of this MTHFD2 response. Knockdown of *MTHFD2* decreased intracellular glycine and increased the intracellular serine concentrations (Fig. 4d), suggesting that the de novo mitochondrial glycine synthesis is highly active in endothelial cells and more important than cellular uptake or cytosolic glycine synthesis. Importantly, oxPAPC decreased the intracellular glycine level, even though it induced the expression of *MTHFD2* (Fig. 4d).

### Regulatory pathways contributing to MTHFD2 expression

Amino acid metabolic reprogramming was not the only response activated by oxPAPC and *MTHFD2* siRNA. The amino acid cluster, the *MTHFD2* Bayesian network and the *MTHFD2* RNAseq signature were also enriched for mTOR and amino acid deprivation and ER stress (Supplementary Fig. 3). Nrf2 signaling was also activated as oxPAPC increases ROS in endothelial cells^16^. Interestingly, the ROS scavenger *N*-acetylcysteine (NAC) but not glycine supplementation prevented the oxPAPC-mediated induction of *MTHFD2* (Fig. 4e). Induction of *MTHFD2* by the activator of the unfolded protein response tunicamycin was affected neither by glycine nor by Nrf2. Nrf2 orchestrates the cellular redox-defense system and was shown to be activated by oxidized phospholipids^17^. The Nrf2 activator *tert*-butylhydroquinone (*t*BHQ) indeed induced *MTHFD2* expression, suggesting that both compounds act in part through similar cascades (Fig. 4f). Indeed, the Nrf2 inhibitor ML385 prevented oxPAPC-mediated induction of *MTHFD2* expression (Fig. 4g). However, whereas the Nrf2 target gene glutathione synthesizing enzyme Glutamate-Cysteine Ligase Modifier Subunit (*GCLM*)^17^ was induced by oxPAPC in the presence or absence of fetal calf serum (FCS) in the culture medium, *MTFHD2* induction was massively attenuated upon serum addition (Supplementary Fig. 4a−c). This suggests that ingredients of the serum satisfy the cellular needs of substrates otherwise generated by the *MTFHD2* network. The *MTHFD2* network was also enriched for mTOR signaling. Application of the mTOR inhibitor rapamycin also inhibited expression of *MTHFD2* and genes of the *MTHFD2* network in HAEC (Supplementary Fig. 4d–f). Indeed, oxPAPC activated mTOR signaling as assessed from phosphorylated ribosomal protein S6 (S6) (Fig. 4h, i) and inhibition of mTOR signaling by torin also prevented oxPAPC-mediated MTHFD2 induction (Fig. 4g). Since both, Nrf2 and mTOR activate the transcription factor *ATF4*^18^, we tested whether the oxPAPC-mediated *MTHFD2* expression was *ATF4* dependent. *ATF4* siRNA indeed partially prevented the oxPAPC-mediated induction of *MTHFD2* (Fig. 4j, Supplementary Fig. 4g) and overexpression of ATF4 increased *PHGDH* and *MTHFD2* expression (Supplementary Fig. 4h−k). Thus, oxPAPC induced *MTHFD2* expression by a complex response in part mediated by *ATF4* comprising redox as well as mTOR signaling.

### OxPAPC elicit endothelial purine nucleotide release

**Fig. 5:**
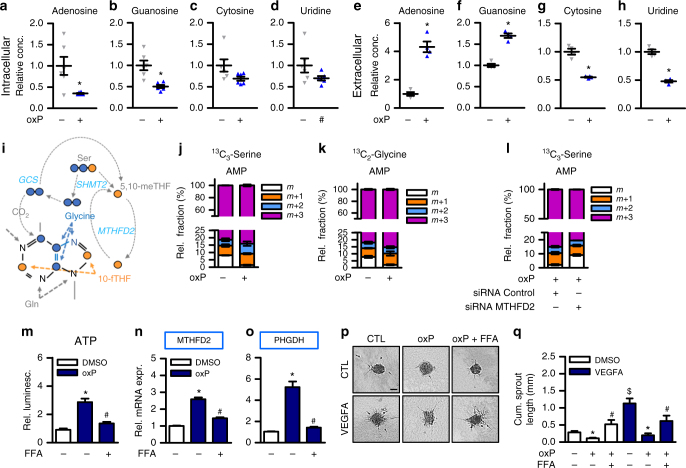
OxPAPC elicits ATP release and inhibition of ATP release prevents induction of *MTHFD2*. **a−d** Nucleoside measurement in HAEC exposed to medium (1% FCS) with (oxP) or without (Ct) oxPAPC for 24 h. Cell lysates were measured by mass spectrometry (*n* = 6). (*p ≤ 0.05 Student’s *t* test). **e−h** Nucleoside measurement in supernatants of HAEC exposed to medium (1% FCS) with or without oxPAPC for 24 h. Supernatants were measured by mass spectrometry (*n* = 4) (*p ≤ 0.05 Student’s *t*-test) **i** Scheme of flow of serine- and glycine-derived carbons which can be incorporated into the purine backbone. **j**, **k** HAEC were treated with ^13^C_3_-serine (**j**) or ^13^C_2_-glycine (**k**) and oxPAPC or control for 24 h and supernatants were measured by mass spectrometry (*n* = 3). Relative fractions of extracellular AMP containing no (*m*), one (*m* + 1), two (*m* + 2) or three (*m* + 3) heavy carbons are shown. l 24 h flux analysis with ^13^C_3_-serine labeling in HAEC with or without siRNA mediated knockdown of *MTHFD2* (*n* = 3). **m** ATP measurement of supernatants of HAEC exposed to medium (1% FCS) with or without oxPAPC and flufenamic acid (FFA, 50 µM) for 8 h. ATP was measured by luminescence and normalized to intracellular RNA concentration (*n* = 7). **n**, **o** qRT-PCR detection of *MTHFD2* and *PHGDH* in HAEC exposed to medium (1% FCS) with or without oxPAPC and flufenamic acid (FFA, 50 µM) for 24 h (*n* = 5). **p**, **q** Spheroid outgrowth assay (**p**) and quantification (**q**) of the cumulative sprout length of HUVEC treated with combinations of oxPAPC, flufenamic acid (FFA, 50 µM) and VEGF-A165 (10 ng ml^−1^) as indicated (*n* = 6). Scale bar: 50 µM. Data are represented as mean ± SEM, *p ≤ 0.05 (oxP vs Ct), ^#^*p* ≤ 0.05 (inhibitor present vs absent), ^$^*p* ≤ 0.05 (VEGFA vs Ct), (ANOVA with Bonferroni post-hoc test if not otherwise indicated)

Why do endothelial glycine levels decrease upon oxPAPC stimulation? Glycine is an important substrate for numerous pathways including heme synthesis, glutathione synthesis and for protein de novo synthesis but particularly important for purine synthesis. Purines, in the form of ATP and GTP, are essential for the cellular energy homeostasis and for DNA and RNA de novo synthesis. Purines are also important signaling transmitters and endothelial cells release purines as signaling autacoids in response to numerous stimuli including shear stress^19^. Through the activation of purinergic receptors, purines impact on vascular homeostasis, coagulation, inflammation and on the control of vascular tone^20^. Thus, we speculated that oxPAPC induces the release of ATP and other nucleotides and thereby depletes endothelial cells of these molecules. Indeed, exposure to oxPAPC reduced the endothelial intracellular purine but not pyrimidine nucleoside pools (Fig. 5a–d). Moreover, the extracellular degradation products of ATP and GTP, but not of pyrimidines, were increased in response to oxPAPC exposure in the cell culture supernatant of HAEC (Fig. 5e–h). MTHFD2 contributes to purine synthesis in the form of glycine and 10-fTHF: Two carbon units are derived from serine which is converted into glycine and incorporated into the purine backbone (Fig. 5i). Two additional single carbon units are derived from two serine molecules and are incorporated into the purine backbone as 10-fTHF. Additionally, the mitochondrial glycine cleavage system can cleave glycine into CO_2_ and 5,10-meTHF which both can be incorporated into the purine backbone. We therefore analyzed the flux of serine- and glycine-derived carbons in response to oxPAPC by heavy isotope tracing. With and without oxPAPC, labeled serine was detected intracellularly in serine labeled cells and labeled glycine was detected to a lesser extent intracellularly in glycine labeled cells, indicating that HAEC take up serine as well as glycine (Supplementary Fig. 5a, b). Extracellular AMP, a degradation product of ATP, contained roughly 90% heavy carbons in heavy serine labeled as well as in heavy glycine labeled cells (Fig. 5j, k). This indicates that HAEC incorporated imported serine as well as imported glycine into the purine backbone of adenosine nucleotide derivatives which are then released from HAEC. OxPAPC-treated HAEC showed comparatively more heavy labeled extracellular AMP which was decreased after silencing of *MTHFD2* (Fig. 5l). In contrast, the purine-derivatives NAD and inosine showed only about 20% incorporation of heavy serine-derived carbon units (Supplementary Fig. 5c, d) and NAD showed only about 10% incorporation of heavy glycine derived carbons (Supplementary Fig. 5e). The incorporation of heavy carbons was decreased upon oxPAPC treatment. This suggests that serine-glycine metabolism in HAEC contributes to the synthesis of adenosine nucleotide derivatives for release which is enhanced by oxPAPC. We therefore hypothesized that oxPAPC activates HAEC to synthesize purine nucleotides. Conforming to this hypothesis, oxPAPC exposure increased the metabolic activity of HAEC as scored by the mitochondrial oxygen consumption rate (OCR) (Supplementary Fig. 5f).

### Blockade of purine release prevents *MTFHD2* induction

If purine release was indeed the mechanism driving the cellular responses to oxPAPC, blockade of ATP release should attenuate the effects of oxPAPC. Flufenamic acid (FFA) blocks ATP release^21^ and inhibits nucleotide transporter (VNUT)-mediated ATP transport^22^. Indeed, FFA not only blocked the oxPAPC-mediated increase in extracellular purines (Fig. 5m), but also prevented the induction of *MTHFD2* and *PHGDH* in response to oxPAPC (Fig. 5n, o). Similarly, the VNUT inhibitors α-glycyrrhetinic acid and A438079 prevented oxPAPC-mediated ATP release (Supplementary Fig. 5g). Moreover, all inhibitors partially restored sprouting after oxPAPC treatment of human umbilical vein endothelial cells (HUVEC) (Fig. 5p, q, Supplementary Fig. 5h−j). In contrast, glycine alone could not rescue oxPAPC-inhibited sprouting (Supplementary Fig. 5k-m).

### Amino acid deprivation induces the MTHFD2 network

**Fig. 6:**
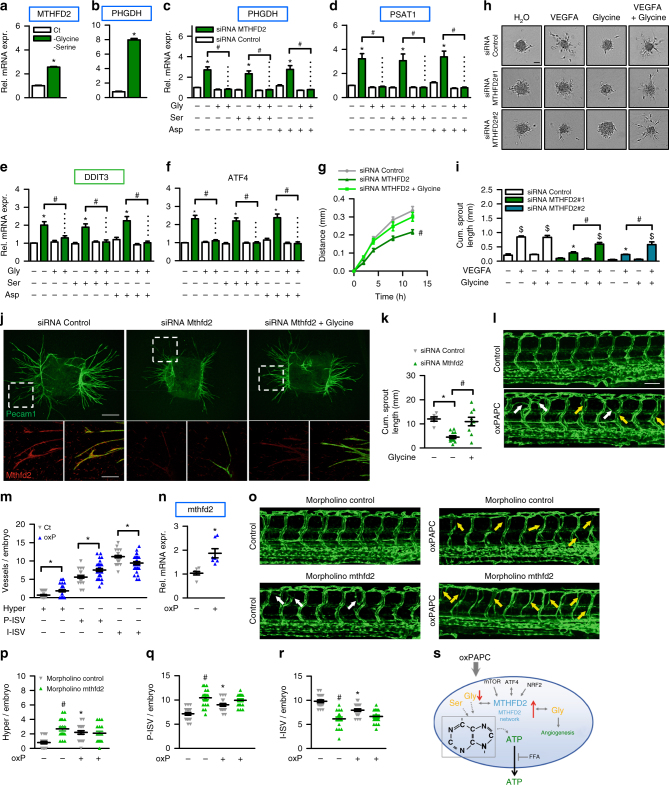
*MTHFD2*-dependent glycine is crucial for angiogenesis. **a**, **b** qRT-PCR detection of *MTHFD2* and *PHGDH* in HAEC treated with (Ct) or without serine (300 µM) and glycine (30 µM) for 16 h (*n* = 4). (*p ≤ 0.05 Student’s *t* test) **c−f** HUVEC treated with the indicated siRNAs were supplemented with or without glycine, serine or asparagine (500 µM) for 16 h (*n* = 8). *DDIT3* DNA damage inducible transcript 3 (also known as CHOP). **g** Scratch wound migration assay of HUVEC treated with the indicated siRNAs and supplemented with or without glycine (500 µM). Migration distance after application of scratch is depicted (*n* = 5). (ANOVA with repeated measures) **h**, **i** Spheroid outgrowth assay (**h**) and quantification of the cumulative sprout length (**i**) of HUVEC treated with or without the siRNAs indicated and VEGF-A165 (10 ng ml^−1^) or glycine (500 µM) (*n* = 6). Scale bar: 50 µM. **j**, **k** Immunofluorescence (**j**) and quantification of cumulative sprout length (**k**) of aortic ring outgrowth in organ culture treated with the indicated siRNAs and glycine (500 µM) (*n* = 6–12). *Pecam1* was used as a marker for endothelial cells. Scale bars: 500 µM (overall image), 100 µM (zoom) **l**, **m** Confocal microscopic image (**l**) of zebrafish vasculature of *tg(fli1:EGFP)* embryos at 72 h post-fertilization (hpf) injected without or with 1 µg µl^−1^ oxPAPC into yolk at 0.5 hpf. White arrows indicate hyperbranches and yellow arrows indicate partial normal intersegmental vessels. Quantitative morphological analysis (**m**) in control (*n* = 30) and oxPAPC (*n* = 31) group shows hyperbranches (Hyper), partial normal (P-ISV) and intact (I-ISV) intersegmental vessels per embryo. The experiment was performed two times. Scale bar: 100 µM. **n** qRT-PCR of zebrafish embryos in **m** normalized to elongation factor 1-alpha (*elfa*) (4–5 embryos pooled per sample) (*n* = 7). **o**–**r** Confocal microscopic image (**o**) and quantification (**p**−**r**) of *tg(fli1:EGFP)* embryos at 72 hpf injected with control (*n* = 20) or mthfd2 morpholino (*n* = 20) at 0.5 hpf with (*n* = 19) and without 1 µg µl^−1^ oxPAPC (*n* = 20). Data are represented as mean ± SEM; **c−i** *p ≤ 0.05 (*MTHFD2* vs Control siRNA), ^#^*p* ≤ 0.05 (with vs without amino acid), ^$^*p* ≤ 0.05 (VEGFA vs Ct) (ANOVA with Bonferroni post-hoc test); **m−r** *p ≤ 0.05 (oxP vs Ct), ^#^*p* ≤ 0.05 (mthfd2 vs control morpholino) (ANOVA with Newman−Keuls post-hoc test). **s** Proposed model of findings

Our observations raise the possibility that the *MTHFD2* network induction in response to oxPAPC is a direct consequence of glycine depletion. To address this, we deprived HAECs of glycine and serine which induced the expression of *MTHFD2* and *PHGDH* (Fig. 6a, b). Additionally, we elicited amino acid deprivation by asparaginase which depletes cells of asparagine^23^ and determined the effect of the histidine analog l-histidinol which inhibits activation of histidine by histidyl-tRNA synthetase^24^. Indeed, both treatments induced the expression of genes in the *MTHFD2* network (Supplementary Fig. 6a, b). These findings suggest that the *MTHFD2* network constitutes an amino acid response that compensates for oxPAPC-induced loss of glycine.

### *MTHFD2*-knockdown-impaired angiogenesis is glycine dependent

In line with this conclusion, we hypothesized that supplementation of glycine should overcome the effects of *MTHFD2* siRNA. Indeed, glycine but not serine or asparagine, fully prevented the expression of the tested genes belonging to the *MTHFD2* network as well as *ATF4* in HUVEC (Fig. 6c–f). To determine the importance of this response we measured endothelial migration in a scratch wound assay and the angiogenic capacity as determined by spheroid outgrowth in response to VEGFA. *MTHFD2* siRNA impaired migration of HUVECs which was prevented by glycine supplementation (Fig. 6g, Supplementary Fig. 6c). Interestingly, also folate but not formate restored normal migration upon knockdown of *MTHFD2* (Supplementary Fig. 6d, e). Impairment of sprouting by silencing *MTHFD2* was prevented by glycine, but not by folate or formate supplementation (Fig. 6h, i, Supplementary Fig. 6f−i). Importantly, proliferation was only slightly decreased upon knockdown of *MTHFD2* and this effect was rescued by glycine whereas oxPAPC inhibited proliferation in an *MTHFD2*-independent manner (Supplementary Fig. 6j). To confirm the importance of *MTHFD2* for angiogenesis, we tested the contribution of *MTHFD2* in an ex vivo organoid mouse model of angiogenesis. Murine aortic rings with siRNA-mediated knockdown of *Mthfd2* showed decreased vessel formation which was restored by glycine supplementation (Fig. 6j, k). Furthermore, zebrafish embryos exposed to oxPAPC showed deregulated vessel formation and increased mRNA expression of *mthfd2* (Fig. 6l–n). Additionally, zebrafish embryos with a morpholino-mediated knockdown of *mthfd2* showed deregulated vasculature morphology (Fig. 6o–r, Supplementary Fig. 6k). Collectively, our findings indicate that oxPAPC induced endothelial glycine depletion; that *MTHFD2* is induced to compensate for glycine deprivation; and that the MTHFD2-dependent glycine production is essential for endothelial cell function. We suggest that oxidized phospholipids induce a metabolic reprogramming response of endothelial cells which allows for the replenishment of cellular nucleotide pools that become depleted as a consequence of oxPAPC-induced nucleotide release (Fig. 6s).

### *MTHFD2* impacts on plasma metabolites in humans

**Fig. 7:**
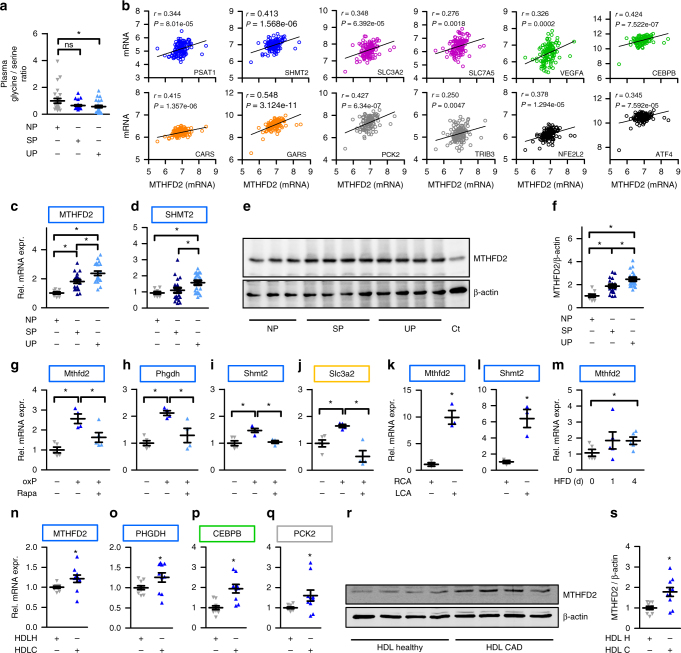
MTHFD2 is deregulated in cardiovascular disease. **a** Glycine to serine ratio in plasma of human subjects with no atherosclerotic plaque (NP) (*n* = 26), stable atherosclerotic plaque (SP) (*n* = 26), and unstable atherosclerotic plaque (UP) (*n* = 26) as assessed by mass spectrometry. **b** Scatter plots showing expression correlation in 126 human carotid plaque samples between *MTHFD2* and genes of the *MTHFD2* network (colored according to Fig. 3a) as well as Nrf2 (*NFE2L2*) and *ATF4* as calculated by Pearson correlation. **c**, **d** Relative mRNA expression of *MTHFD2* and *SHMT2* in plaque material of human subjects with unstable atherosclerotic plaque (UP) (*n* = 20), stable atherosclerotic plaque (SP) (*n* = 20), or non-atherosclerotic artery (NP) (*n* = 8) (normalized to *18SrRNA*) (*n* = 8). **e**, **f** Western blot analysis of MTHFD2 expression (**e**) and quantification (**f**) of plaque material from human subjects with unstable atherosclerotic plaque (SP) (*n* = 20), stable atherosclerotic plaque (SP) (*n* = 20), or non-atherosclerotic artery (NP) (*n* = 8). **g**–**j** Relative mRNA expression of *Mthfd2*, *Phgdh*, *Shmt2*, and *Slc3a2* in mouse thoracic aortic rings kept in organ culture and exposed to medium (1% FCS) with or without oxPAPC and rapamycin as indicated for 8 h (normalized to *18SrRNA*) (*n* ≥ 4). **k**, **l** Relative mRNA expression of *Mthfd2* and *Shmt2* in the endothelium of partially ligated left carotid artery (LCA) compared to healthy right carotid artery (RCA) 48 h post ligation (normalized to *18S rRNA*) (*n* = 3). (*p ≤ 0.05 Student’s *t* test). **m** Relative mRNA expression of *Mthfd2* in the endothelium of the left carotid artery of *ApoE−/−* mice which were fed with high fat diet (HFD) for 0, 1, or 4 days (normalized to *18S rRNA*) (*n* = 5). **n**–**q** Relative mRNA expression of *MTHFD2*, *PHGDH*, *CEBPB* and *PCK2* in HAEC exposed to HDL from healthy human subjects (*n* = 10) or human subjects with CAD (*n* = 10) for 4 h. (*p ≤ 0.05 Mann Whitney test). **r**, **s** Western blot analysis of MTHFD2 (**r**) and quantification (**s**) of HAEC treated as in **f** for 24 h (*n* = 10) (*p ≤ 0.05 Mann Whitney test). Data are represented as mean ± SEM, *p ≤ 0.05 (ANOVA with Newman−Keuls post-hoc test if not otherwise indicated)

To support the role of the *MTHFD2* network and serine-glycine metabolism, we speculated that alterations of the *MTHFD2* network might impact on the plasma concentrations of relevant metabolites in humans. It should be mentioned that this approach would not allow us to link the findings to any specific vascular alterations. We obtained data from a human genome-wide association study that provides the plasma abundance of 400 metabolites^25^ allowing us to test whether genes in the *MTHFD2* network were associated with the metabolite abundances. For this, we searched for SNPs (meta-analysis *p* value < 1×10^−4^) significantly associated with human plasma metabolites. An SNP rs10174907 in *MTHFD2* was significantly associated with plasma *N*-acetylglycine concentration, SNPs associated with *N*-acetylglycine concentration were enriched for the genes in the *MTHFD2* network (FET *p* value < 0.0045) and genes of the *MTHFD2* network showed associations with plasma metabolites directly or indirectly related to glycine metabolism (Table 1). In total, 60 SNPs in 28 genes in the *MTHFD2* network were associated with plasma metabolite concentrations (Supplementary Data 4). This analysis suggests that the *MTHFD2* network, as constructed by us for HAECs, is essential in the regulation of plasma metabolite concentrations. To address a link to vascular disease, the plasma glycine to serine ratio in a human cohort with carotid artery disease was determined. Interestingly, the plasma glycine to serine ratio was decreased in subjects with unstable atherosclerotic plaques (*n* = 26) and decreased by tendency in subjects with stable atherosclerotic plaques (*n* = 26) compared to plaque-free subjects (*n* = 26) (Fig. 7a).

**Table 1:** Association between *MTHFD2* network gene loci and plasma metabolites directly or indirectly associated with glycine metabolism

Gene	Metabolite	SNP	*p* value
*MTHFD2*	*N*-acetylglycine	rs10174907	1.43E-05
*EPAS1*	*N*-acetylglycine	rs1530628, rs2197698, rs1530627	8.69E-05, 9.45E-05, 9.76E-05
*GLDC*	Glycine	rs2297442	2.94E-05
*AARS*	Tyrosine	rs9936903	1.89E-08
*LTA4H*	Methionine	rs12579455	4.99E-05
*SETX*	Citrulline	rs612169, rs545971, rs674302, rs514659	5.91E-05, 6.35E-05, 6.39E-05, 6.86E-05
*EPAS1*	Tryptophane	rs1530628	8.13E-05
*PCK2*	Glycerol 3-phosphate	rs1062230	2.60E-05

### MTHFD2 expression is increased in cardiovascular diseases

Since atherosclerosis is driven in part by oxidized lipids, we hypothesized that the *MTHFD2* network is active in human atherosclerotic samples. To identify whether the *MTFHD2* network has any association with cardio-vascular disease we obtained the data set of the CARDIoGRAMplusC4D genome-wide association (GWAS) study^26^. For our analysis, we searched for SNPs (*p* value from logistic regression <1×10^−4^) significantly associated with CAD and identified that genes of the *MTHFD2* network contained several CAD-associated SNPs (Table 2, Supplementary Data 5). Moreover, the CAD risk loci were enriched for genes of the *MTHFD2* network (FET *p* value < 0.0077). Collectively, the data raise the possibility that the *MTHFD2* network contributes to CAD development.

**Table 2:** Association between genes within the *MTHFD2* network and CAD risk loci

Gene	SNP	*p* value
*SORT1*	rs7528419, rs12740374, rs4970834, rs611917	7.05E-08, 1.25E-07, 1.04E-06, 1.45E-06
*SLC7A1*	rs9551751	7.93E-08
*SHMT2*	rs11172113	1.72E-06
*DDIT3*	rs11172113	1.72E-06
*MARS*	rs11172113	1.72E-06
*CEBPB*	rs6095611, rs1034056, rs6067199, rs6091031	9.14E-06, 1.38E-05, 1.59E-05, 1.75E-05
*FOXO1*	rs9594389, rs7323896	1.68E-05, 1.78E-05
*GJA4*	rs1336624	2.42E-05
*CDC42EP2*	rs12419237	4.82E-05
*PIK3C2B*	rs16854023	5.57E-05

To address whether the expression of the *MTHFD2* network is changed in human atherosclerosis, we reanalyzed data from a study comparing carotid artery gene expression between 32 healthy and 32 plaque-carrying specimens (GSE43292)^27^. Expression changes of the *MTHFD2* network genes in the carotid arteries in response to atherosclerosis were similar to those of endothelial cells in response to oxPAPC (Supplementary Fig. 7a). The majority of the *MTHFD2* network genes that were induced in response to oxPAPC were also increased in atherosclerosis. Moreover, an association was also observed for oxPAPC- and atherosclerosis-mediated gene repression. In line with this observation, expression of *MHTFD2* in 126 human carotid plaque samples of the BiKE study (GSE21545)^28^ was positively correlated with genes of the *MTHFD2* network as well as Nrf2 and *ATF4* but not with *MTHFD1L* which does not belong to the *MTHFD2* network (Fig. 7b, Supplementary Fig. 7b).

To correlate our findings with late stage atherosclerosis, we tested carotid endo-atherectomy specimens from the same human cohort as in Fig. 7a (patient characteristics, Supplementary Table 7). The tissue samples were classified as stable plaques (*n* = 20) or unstable plaques (*n* = 20). *SHMT2* expression was increased in plaque material of subjects with unstable plaques and *MTHFD2* expression was increased in plaque material of both subpopulations (stable and unstable plaques) compared to healthy arteries (Fig. 7c, d). Conforming to the mRNA expression pattern, MTHFD2 expression was increased in vessels of subjects with atherosclerotic disease as compared to subjects without plaque (Fig. 7e, f). Subjects with unstable plaques exhibited a significantly decreased plasma glycine to serine ratio and had a stronger increase in MTHFD2 expression than subjects with stable plaques. Unstable plaques are inflammatory activated but are also enriched in endothelial cells compared to stable plaque and thus are characterized by increased angiogenesis. Taken together, these data support the notion that MTHFD2 is expressed and upregulated in human atherosclerosis and seems to affect the amino acid glycine and serine metabolism.

A problem of these human studies, however, is that the analyses focused on a rather late time point-established vascular disease in a heterocellular context (whole organ) whereas our initial experiments were carried out in endothelial cells at an early time point. To bridge this gap, we returned to mouse models. Exposure of the murine thoracic aorta to oxPAPC in organ culture increased the expression of the network genes *Mthfd2*, *Phgdh*, *Shmt2* and *Slc3a2*, an effect prevented by rapamycin (Fig. 7g–j). Moreover, the *MTHFD2* network was induced in endothelial cells harvested from carotid arteries in the partial ligation model, an in vivo mouse model of blood flow withdrawal-induced accelerated atherosclerosis^29^. *Mthfd2* and *Shmt2* were significantly induced in the endothelium of partially ligated arteries compared to non-ligated arteries (Fig. 7k, l). Importantly, endothelial expression of genes within the *MTHFD2* network was already induced 24 and 48 h after partial ligation in a previously published data set^29^ (Supplementary Fig. 7c).

These findings may suggest that the *MTHFD2* network induction is fundamental to the atherosclerotic disease at large and not only observed upon direct stimulation with oxPAPC. To test this hypothesis, we subjected *ApoE−/−* mice to a brief course of an atherogenic high-fat Paigen diet. Paigen diet is a specific high fat diet that contains not only triacylglycerides (15.5%) and cholesterol (1.25%) but also cholate (0.5%). When administered, high plasma lipid and cholesterol levels, as well as systemic inflammation, are induced and massively accelerate atherosclerosis development. *ApoE−/−* mice are particularly sensitive to this diet as these mice already exhibit high VLDL levels under basal conditions^30^. Importantly, already 4 days after the initiation of the diet, the expression of *Mthfd2* was increased in the endothelium of the murine carotid artery (Fig. 7m). Encouraged by this finding, we wondered whether also atherogenic lipoproteins obtained from human subjects with vascular disease would induce the *MTHFD2* network in endothelial cells. To study this, we obtained high-density lipoproteins (HDL) from healthy subjects (*n* = 10) or stable CAD patients (*n* = 10) (patient characteristics, Supplementary Table 8). It was previously established that HDL from CAD patients loses its protective effects and becomes atherogenic^31^. Indeed, HDL derived from CAD patients compared to HDL from healthy subjects induced the network genes *MTHFD2*, *PHGDH*, *CEBPB*, and *PCK2* in HAEC (Fig. 7n–q). This effect was sufficiently strong to translate into protein expression: As determined by western blot analysis, MTHFD2 protein abundance was increased in HAEC exposed to CAD patient-derived HDL (Fig. 7r, s).

## Discussion

Using an integrative network modeling approach, we identified an unexpected association of atherogenic lipids and atherosclerosis with the mitochondrial one-carbon metabolism. In human endothelial cells, oxidized phospholipids activated a network in which *MTHFD2* was identified as a key driver to increase the generation of glycine. Glycine was required for the formation of purines to compensate for their loss from endothelial cells in response to oxidized phospholipids.

Endothelial metabolism has been recognized as an important determinant of endothelial cell function^32^, controlling angiogenesis and the adaptation of endothelial cells to different environmental conditions^33^. Oxidized phospholipids, however, have not yet been linked to endothelial metabolism. Deregulated amino acid metabolism and in particular overexpression of serine-glycine-synthesizing enzymes and mitochondrial one-carbon metabolism is associated with rapid proliferation of cancer cells^34^. Thus, MTHFD2 is highly expressed in many cancers or the embryo^35^, whereas expression is low in most postmitotic cells^12,36^. We show that MTHFD2 contributes to glycine synthesis in endothelial cells in a disease-relevant manner. Plasma glycine is inversely associated with myocardial infarction risk^37^ and in our present work we found an association between plasma amino acids, the *MTHFD2* genotype and cardiovascular risk. Moreover, knockdown of *MTHFD2* reduced the angiogenic function of cultured cells. Importantly, glycine prevented this effect and also blocked *MTHFD2* network induction in response to *MTHFD2* siRNA. Currently, MTHFD2 is tested as a potential drug target for cancer therapy^38^. Our data suggest that MTHFD2 inhibition might also attenuate tumor angiogenesis.

MTHFD2 contributes to NADPH production that is required for nucleic acid and lipid synthesis and to glutathione and thioredoxin recycling^39^. Glycine is also needed for glutathione synthesis. In line with this, the *MTHFD2* network comprised genes responsive to amino acid starvation, ER stress, mTORC1, and Nrf2 activation. This response pattern reflects the central position of glycine in cellular metabolism.

Purines are products of glycine in the intermediate metabolism and induction of the *MTHFD2* network facilitated the endothelial production of purines in response to oxPAPC. In fact, purine nucleotides are important autacoids which, if released from endothelial cells, elicit a plethora of responses. The local release of purine nucleotides from atherosclerotic plaques and the subsequent activation of cellular purinergic receptors is thought to promote the development of atherosclerosis^40^. Nucleotide release is an endothelial stress reaction and occurs in response to thrombin, shear stress, and oxidative stress induced by copper-oxidized LDL^19,41,42^. The mechanism of stress-induced purine nucleotide release is not fully understood but a role of gap junctions has been suggested^41,43^. Indeed, gap junction blockers prevented ATP release in response to oxPAPC in the present study. The compounds also prevented the induction of the *MTHFD2* network in response to oxPAPC suggesting that the induction of the network is a compensatory reaction to the loss of purines.

OxPAPC and knockdown of *MTHFD2* induced *ATF4* and several ER stress response genes like *DDIT3* and *sXBP1* which were also members of the *MTHFD2* network. This observation is in line with the fact that ER stress is present in atherosclerotic plaques and promotes the atherosclerotic process through apoptosis^44^. Important pro-atherosclerotic lipids like 7-ketocholesterol through this mechanism fuel the atherosclerotic process^45^. ER stress is also present in regions of the arterial endothelium susceptible to atherosclerosis^46^. This and the inflammatory activation of the endothelium in the atherosclerotic process explain why we observed an activation of the *MTHFD2* network in atherosclerotic plaques. Moreover, our finding that decreasing *MTHFD2* expression induces endothelial cell dysfunction could serve as an explanation of why genetic variations in genes of the MTHFD2 network were associated with CAD risk.

Oxidation of lipoproteins which has been best studied for LDL occurs in the intima of the arterial wall and thus oxLDL is scarcely found in the plasma^47^. Nevertheless, we observed that the highly inflammatory and atherogenic lipid- and cholate-rich Paigen diet in *ApoE−/−* mice as well as HDL particles obtained from the plasma of patients with CAD increased endothelial *MTHFD2* expression. Although these data link *MTHFD2* to the development of atherosclerosis, they must not raise the impression that this response is mediated through oxPAPC. As we observed in the present study, *MTHFD2* is a highly stress-sensitive gene and induced by numerous pathways. In fact, the dysfunctional HDL from CAD patients induces endothelial oxidative stress^31^ and also any high cholesterol diet increases endothelial ROS formation^48^. Thus, although the present work on oxPAPC-mediated induction of *MTHFD2* was instrumental in linking signaling of oxidized lipids to amino acid metabolism in endothelial cells, the findings extend beyond this pathway: Endothelial cell activation in general appears to induce *MTHFD2* which is a meaningful response given that ATP release is a stereotypic response of endothelial cells to stress.

In conclusion, by taking advantage of an integrative network modeling approach we unveiled a new reaction pattern of endothelial cells. Our work not only demonstrates the power of Bayesian network analysis to uncover novel signaling mechanisms in vascular disease, it also illustrates the enormous secretory capacity of endothelial nucleotide release in the signaling context. It appears that endothelial cells prioritize autacoid release to such an extent that they would rather become deprived of vital metabolic components than neglect their signaling function.

## Materials and methods

### Reagents

The following reagents were used: NAC (Sigma-Aldrich, Cat# A7250), tunicamycin (Enzo Life Sciences, Cat# BML-CC104), glycine (VWR, Cat# 10119CU), *t*BHQ (Sigma-Aldrich, Cat# 19986), ML385 (Sigma-Aldrich, Cat# SML1833), Rapamycin (Sigma-Aldrich, Cat# R0395), Torin 2 (Selleckchem, Cat# S2817), serine (Sigma-Aldrich, Cat# S4500), asparagine (Sigma-Aldrich, Cat# A4159), VEGF-A 165 (R&D, Cat# 293-VE), Asparaginase (Sigma-Aldrich, Cat# A3809), l-Histidinol (Sigma-Aldrich, Cat# H6647), FFA (Sigma-Aldrich, Cat# F9905), α-glycyrrhetinic acid (Sigma-Aldrich, Cat# G10105), A438079 (Sigma-Aldrich, Cat# A9736), Oligomycin A (Sigma-Aldrich, Cat# 75351), CCCP (Sigma-Aldrich, Cat# C2759), antimycin A (Sigma-Aldrich, Cat# A8674), rotenone (Sigma-Aldrich, Cat# R8875), formate (Sigma-Aldrich, Cat# 06473), folate (Enzo Life Sciences, Cat# ALX-460-006). The following antibodies were used: pS6 (Cell Signaling, Cat# #2215, dilution 1:1000), S6 (Cell Signaling, Cat# 2317, dilution 1:1000), MTHFD2 (Proteintech, Cat# 12270-1-AP, WB dilution 1:1000, IF dilution 1:200), SHMT2 (Bethyl, Cat# A305-351A, dilution 1:2000), β-actin (Cell Signaling, Cat# 4970S, dilution 1:1000), Pecam-1 (BD Biosciences, Cat# 553370, dilution 1:200), FLAG (Sigma-Aldrich, Cat# F3165, dilution 1:1500). Primers for qRT-PCR are listed in Supplementary Table 9.

### Gene set enrichment analysis

Genes of interest (e.g. genes within each subnetwork or differential connectivity cluster compared to whole network or all differential connectivity clusters) compared against a collection of canonical and non-canonical gene sets (MSigDB v6.0)^49^ by using Fisher’s exact test in R (*p* values correspond to Fisher’s exact test). Overlaps between the *MTHFD2* RNAseq signature, the amino acid cluster, and the MTHFD2 network were computed using Fisher’s exact test in R.

### RNA sequencing and data analysis

RNA isolated from HAEC was treated with DNase (Qiagen, Cat# 79254). Library construction (LncRNA library, Ribo-Zero), quality assessment, and sequencing (HiSeqSE50) were performed by Novogene. Differentially expressed genes were identified using the following procedure: firstly, sequencing reads were aligned to the human reference genome hg19 using TopHat^50^. Next, the mapped sequences were aligned with htseq-count to quantify the read count for each gene^51^. The edgeR package^52^ was then used to identify differentially expressed genes between control and siRNA treatment. We detected 399 differentially expressed genes at FDR of 0.05 (Benjamini−Hochberg).

### Differential connectivity clusters

Differentially co-expressed gene pairs and differential connectivity (DC) clusters were computed as the following: In short, Spearman correlation coefficients of each gene pair were computed and transformed into Fisher’s Z-statistics^9^. Next, Q statistics were computed and 997 permutations were conducted by randomly assigning sample labels. A final cutoff of *Q*_0_ = 22.5 that corresponds to FDR = 4.89% was used to detect DC pairs. In addition a given gene pair had to be significantly co-expressed (Spearman’s correlation *p* value< 0.01) in either control or oxPAPC group to be called a differentially co-expressed DC gene pair. If the differentially co-expressed gene pair was significantly co-expressed in control group but not oxPAPC group it was assigned to the LOC category and if the DC gene pair was significantly co-expressed in oxPAPC but not control group it was assigned to the GOC category.

### HAEC Bayesian networks

The accuracy of Bayesian networks is highly dependent on the number of samples used in the network construction process. We showed in a series of simulation studies that accuracy of reconstructed networks significantly improves when sample size increases from 100 to 1000^53^. In the same paper, we showed that integration of genetic information as structure prior can significantly improve accuracy of constructed Bayesian networks when sample size is between 100 and 300. Experimentally, we systematically validated networks constructed with genetic priors and inferred key drivers in yeast^7^, mouse^54^ and human^55^ with sample sizes in the above range. In this study, we constructed HAEC Bayesian networks based on 147 samples with genetic data integrated as priors.

Bayesian networks are directed acyclic graphs in which the edges of the graph are defined by conditional probabilities that characterize the distribution of states of each node given the state of its parents. The network topology defines a partitioned joint probability distribution over all nodes in a network, such that the probability distribution of states of a node depends only on the states of its parent nodes: formally, a joint probability distribution $p(X)$ on a set of nodes $X$ can be decomposed as $p(X) = \mathop {\prod}\nolimits_i {p(X^i} |{\mathrm{Pa}}(X^i))$, where ${\mathrm{Pa}}(X_{}^i)$ represents the parent set of $X_{}^i$. In our networks, each node represents transcription expression of a gene. These conditional probabilities reflect not only relationships between genes, but also the stochastic nature of these relationships, as well as noise in the data used to reconstruct the network.

Bayes formula allows us to determine the likelihood of a network model $M$ given observed data $D$ as a function of our prior belief that the model is correct and the probability of the observed data given the model: *P*(*M|D*) *~* *P*(*D|M*)*P(*M*). The number of possible network structures grows super-exponentially with the number of nodes, so an exhaustive search of all possible structures to find the one best supported by the data is not feasible, even for a relatively small number of nodes. We employed Monte Carlo Markov Chain (MCMC)^56^ simulation to identify potentially thousands of different plausible networks, which are then combined to obtain a consensus network. Each reconstruction begins with a null network. Small random changes are then made to the network by flipping, adding, or deleting individual edges, ultimately accepting those changes that lead to an overall improvement in the fit of the network to the data. We assess whether a change improves the network model using the Bayesian Information Criterion (BIC)^57^ which avoids overfitting by imposing a cost on the addition of new parameters. This is equivalent to imposing a lower prior probability $P(M)$ on models with larger numbers of parameters.

Even though edges in Bayesian networks are directed, we can’t infer causal relationships from the structure directly in general. For example, in a network with two nodes, $X_{}^{\mathrm{1}}$ and $X_{}^{\mathrm{2}}$, the two models $X_{}^{\mathrm{1}} \to X_{}^{\mathrm{2}}$ and $X_{}^{\mathrm{2}} \to X_{}^{\mathrm{1}}$ have equal probability distributions as $p(X^{\mathrm{1}}{\mathrm{,}}X^{\mathrm{2}}) = p(X^2{\mathrm{|}}X^1)p(X^1) = p(X^1{\mathrm{|}}X^{\mathrm{2}})p(X^{\mathrm{2}})$. Thus, by data itself, we can’t infer whether $X_{}^{\mathrm{1}}$ is causal to $X_{}^{\mathrm{2}}$, or vice versa. In a more general case, a network with three nodes, $X_{}^{\mathrm{1}}$, $X_{}^{\mathrm{2}}$, and $X_{}^3$, there are multiple groups of structures that are mathematically equivalent. For example, the following three different models, ${\mathrm{M}}1:X^1 \to X^2,X^2 \to X^3$, ${\mathrm{M2}}:X^2 \to X^1,X^2 \to X^3$, and ${\mathrm{M3}}:X^2 \to X^1,X^3 \to X^2$, are Markov equivalent (which means that they all encode for the same conditional independent relationships). In the above case, all three structures encode the same conditional independent relationship, $X^1 \not \! \bot X^3\left| {X^2} \right.$, $X^1$ and $X^3$ are independent conditioning on $X^2$, and they are mathematically equal

$$\begin{array}{l}p(X) = p({\mathrm{M}}1|D) = p(X^2|X^1)p(X^1)p(X^3|X^2)\\ {\kern 1pt} {\kern 1pt} {\kern 1pt} {\kern 1pt} {\kern 1pt} {\kern 1pt} {\kern 1pt} {\kern 1pt} {\kern 1pt} {\kern 1pt} {\kern 1pt} {\kern 1pt} {\kern 1pt} {\kern 1pt} {\kern 1pt} {\kern 1pt} {\kern 1pt} {\kern 1pt} {\kern 1pt} {\kern 1pt} {\kern 1pt} = p({\mathrm{M2}}|D) = p(X^1|X^2)p(X^2)p(X^3|X^2)\\ {\kern 1pt} {\kern 1pt} {\kern 1pt} {\kern 1pt} {\kern 1pt} {\kern 1pt} {\kern 1pt} {\kern 1pt} {\kern 1pt} {\kern 1pt} {\kern 1pt} {\kern 1pt} {\kern 1pt} {\kern 1pt} {\kern 1pt} {\kern 1pt} {\kern 1pt} {\kern 1pt} {\kern 1pt} {\kern 1pt} {\kern 1pt} = p({\mathrm{M3}}|D) = p(X^2|X^3)p(X^3)p(X^1|X^2)\end{array}.$$

Thus, we can’t infer whether $X^1$ is causal to $X^2$ or vice versa from these types of structures. However, there is a class of structures, V-shape structure (e.g. ${\mathrm{Mv}}:X^1 \to X^2,X^3 \to X^2$), which has no Markov equivalent structure. In this case, we can infer causal relationships. There are more parameters to estimate in the Mv model than M1, M2, or M3, which means a large penalty in BIC score for the Mv model. In practice, a large sample size is needed to differentiate the Mv model from the M1, M2, or M3 models.

### Incorporating genetic data as a structure prior in the HAEC Bayesian networks

In general, Bayesian networks can only be solved to Markov equivalent structures, so that it is often not possible to determine the causal direction of a link between two nodes even though Bayesian networks are directed graphs. However, the Bayesian network reconstruction algorithm can take advantage of the experimental design by incorporating genetic data to break the symmetry among nodes in the network that lead to Markov equivalent structures, thereby providing a way to infer causal directions in the network in an unambiguous fashion^58^. We modified the reconstruction algorithm to incorporate eSNP data as prior as following: genes with *cis*-eSNP^59^ are allowed to be parent nodes of genes without *cis*-eSNPs, but genes without *cis*-eSNPs are not allowed to be parents of genes with *cis*-eSNPs, $p(\rm trans - > \, cis) = 0$. We have shown that integrating genetic data such as *cis*-acting eSNP or eQTLs (excluding edges into certain nodes) improves the quality of the network reconstruction by simulations^53^ and by experimental validations^7,58^. We note that in applying this particular version of the Bayesian network reconstruction algorithm (incorporating genetic information as a prior); if genetic information is not available or is ignored, the population is simply treated as a population with random genetic perturbations.

### Averaging network models

Searching optimal Bayesian network (BN) structures given a data set is an NP-hard problem. We employed an MCMC method to do local search of optimal structures as described above. As the method is stochastic, the resulting structure will be different for each run. In our process, 1000 BNs were reconstructed using different random seeds to start the stochastic reconstruction process. From the resulting set of 1000 networks generated by this process, edges that appeared in greater than 30% of the networks were used to define a consensus network. A 30% cutoff threshold for edge inclusion was based on our simulation study^53^, where a 30% cutoff yields the best tradeoff between recall rate and precision. The consensus network resulting from the averaging process may not be a BN (a directed acyclic graph). To ensure the consensus network structure is a directed acyclic graph, edges in this consensus network were removed if and only if the edge was involved in a loop and the edge was the most weakly supported of all edges making up the loop.

### Identification of key drivers and associated subnetworks

For each Bayesian network, we further identified the key causal regulators by examining the number of N-hob downstream nodes (NHDN) for each gene in the directed network^53^. For a given network, let *μ* be the numbers of N-hop downstream nodes and *d* be the out degrees for all the genes. The genes with the number of N-hop downstream nodes (NHDN) greater than $\overline \mu + \sigma (\mu )$ are nominated as key drivers. The key drivers with degree above $\overline d + 2\sigma (d)$, where *d* denote the number of downstream genes, become key drivers. These criteria identified genes with number of downstream nodes and number of outlinks significantly above the corresponding average value. Based on the causal networks which we constructed, we identified 29 key drivers in control Bayesian network and 27 key drivers in oxPAPC Bayesian network. The subnetwork associated with each key driver was defined as downstream nodes (i.e. neighbors in outdegree direction) with the key drivers as the seeding point. Edges remained the same as in the complete network. Cytoscape 3.4 was used for network visualization.

### Assessment of Bayesian networks

To assess the accuracy of the HAEC Bayesian networks, we compared the BNs with several widely used databases of gene networks and gene sets: (1) 37,080 interactions covering 9465 genes from Human Protein Reference Database (HPRD) database^60^, (2) 195,859 high confident interactions covering 12,427 genes from STRING database^61^, (3) 1329 canonical pathways covering 8439 genes from MsigDB databases^62^, and (4) 11,174 Gene Ontology (GO) annotation sets (sets with size ≥ 200 are excluded) covering 11,508 genes. In particular, we calculated the percentage of our inferred gene-gene connections that are in existing protein/gene network databases, or within the same pathway in gene set databases. For comparison, we generated 100 random networks for corresponding BNs by using the degree.sequence.game function in the igraph R package, and estimated the accuracy of random networks.

Additionally, we assessed the predictive power of our BNs by using gene sets closely regulated in endothelial cells. In particular, we used two independent gene sets, which include 77 gene sets related with endothelial cell from MsigDB, and 400 siRNA gene signatures in HUVEC^63^. For siRNA gene signatures, we downloaded and preprocessed microarray data as previously described^63^, and genes with a *z*-score >2 and <−2 were defined as significantly upregulated and downregulated genes, respectively. Based on these gene sets, we compared accuracy of our BNs to that of widely used gene networks including HRPD database by calculating the percentage of gene−gene connections that are within the same gene set.

### Human carotid atheroma, Biobank-of-Karolinska-Endarterectomy, and partial carotid artery ligation data

We retrieved human atherosclerotic plaque data^27^, which included 32 paired samples of atheroma plaque and macroscopically intact tissue. The mRNA expression levels were downloaded from Gene Expression Omnibus (GEO) with the corresponding GEO accession ID GSE43292. Each platform’s probe ID was mapped to the corresponding gene symbol and the expression levels were averaged over multiple probes mapped to the same gene symbol. The significance level of differentially expressed genes was calculated by Wilcoxon rank sum test.

Similarly, mRNA expression levels in 126 human carotid plaque samples from the Biobank Each (BiKE) were downloaded (GSE21545)^28^. For correlation analysis of gene expression comparing *MTHFD2* and other genes within the *MTHFD2* network, Pearson correlation coefficient was calculated in R by using the cor.test function.

Our previously published microarray^29^ was reanalyzed for expression levels of genes within the *MTHFD2* network 24, 48 h and 2 weeks after partial carotid artery ligation surgery.

### Genome-wide association studies (GWAS) of plasma metabolites and coronary artery disease

We collected significant SNPs (meta-analysis *p* value < 1×10^−4^) associated with one of more than 400 metabolites in human blood in a genome-wide association study (http://metabolomics.helmholtz-muenchen.de/gwas/)^25^. Candidate SNPs were mapped to genes if their physical locations were within ±5 kb of gene bodies. We also compared candidate SNPs in CARDIoGRAMplusC4D (Coronary ARtery DIsease Genome wide Replication and Meta-analysis (CARDIoGRAM) plus The Coronary Artery Disease (C4D) Genetics) consortium. In particular, we collected significant SNPs with *p* value < 1×10^−4^ based on CARDIoGRAMplus4D 1000 Genome-based GWAS study, which is a meta-analysis of GWAS studies using 1000 genomes with 38 million variants^26^. We searched significant SNPs whose physical location is within ±500 kb of coding region of genes within our MTHFD2 network.

### Cell culture

Human aortic endothelial cells (PeloBiotech, Cat# 304-05a, Lot No. 2366) and pooled HUVEC (Lonza, Cat# CC-2519, Lot No. 0000371074, 0000369146, 0000314457) were cultured in a humidified atmosphere of 5% CO_2_ at 37 °C on gelatin (Sigma-Aldrich, Cat# 61890)-coated dishes. Cells were grown in enhanced endothelial cell growth medium (PeloBiotech, Cat# PB-C-MH-100-2199), consisting of endothelial basal medium (EBM) supplemented with glutamine, bFGF, hEGF, VEGF from this kit and 8% FCS (Biochrom, Cat# S0113), penicillin (50 U ml^−1^) and streptomycin (50 µg ml^−1^) (ThermoFisher, Cat# 15140-122). HUVEC were used at passages 3−5. HAEC were used at passages 3–10. During oxPAPC treatment cells were starved in EBM containing 1% FCS. For glycine and serine starvation, RPMI-1640 media without glucose, glycine and serine (Teknova, Cat# R9660-02) was used and supplemented with 1 g l^−1^ glucose (Sigma-Aldrich, Cat# 16301). As primary cells were used at low passage, myoplasm testing was not performed.

### OxPAPC treatment

OxPAPC was either derived from Invivogen (Cat# tlrl-oxp1) or Avanti Polar Lipids (Cat# 870604) or produced from PAPC (Avanti Polar Lipids, Cat#, 850459) by 24–74 h autoxidation of the nitrogen-air-dried PAPC-lipid film. OxPAPC was dissolved in EBM containing 1% FCS and cells were treated in EBM containing 1% FCS. Since the activity of oxPAPC varies, concentrations in the range of 40–65 µg ml^−1^ were used. Where indicated low oxPAPC refers to 40–50 µg ml^−1^ and high oxPAPC refers to 50–65 µg ml^−1^.

### SiRNA transfection and overexpression and proliferation

For siRNA treatment, endothelial cells (80–90% confluent) were transfected with Lipofectamine RNAiMAX transfection reagent (Thermo Fisher, Cat# 13778150) according to the instructions provided by Thermo Fisher. The target-specific siRNAs for *MTHFD2* and *PSAT1* were obtained from Invitrogen (Stealth RNAi, Cat# 1299001) and for *ATF4* from Sigma-Aldrich (Cat# NM_001675). The scrambled siRNAs were obtained from Invitrogen (Stealth RNAi, siRNA Negative Control Med GC Duplex). Plasmid overexpression in endothelial cells was performed with the Neon electroporation system (Invitrogen) with FLAG-tagged *ATF4* (gift from Yihong Ye, Addgene plasmid pRK-ATF4 #26114)^64^. Cells were lysed 24 h after electroporation. For proliferation assay cells were counted 48 h after transfection with a CASY cell counter (OMNI Life Sciences).

### RNA isolation and qRT-PCR in endothelial cells

Total RNA isolation was performed with an RNA Mini Kit (Bio&Sell, Cat# BS67.311). Reverse transcription was done with SuperScript III Reverse Transcriptase (Thermo Fisher, Cat# 18080044) and oligo(dT) (Sigma-Aldrich, Cat# O4387) together with random primers (Promega, Cat #C118A). CopyDNA amplification was measured with qRT-PCR using SYBR Green Master Mix and ROX as reference dye (BioRad, Cat# 172-5125) in a PikoReal cycler (ThermoFisher). Relative expression of target genes was, if not otherwise indicated, normalized to β-Actin and analyzed by the delta−delta Ct method with the PikoReal software (ThermoFisher). In siRNA experiments RNA was isolated 48–72 h after transfection.

### Protein isolation and western analyses in endothelial cells

Cells were washed two times with Hanks solution (Applichem) and lysed with RIPA lysis buffer supplemented with Benzonase (Sigma-Aldrich, Cat# E1014), sodium orthovanadate (Applichem, Cat# A2196), PhosStop (Sigma-Aldrich, Cat# PHOSS-RO), Beta-glycerophosphate (Sigma-Aldrich, Cat# G9422), Sodium fluoride (Sigma-Aldrich, Cat# S7920) for 5–10 min. Cell extracts were boiled in Laemmli buffer, equal amounts of protein were separated with SDS-PAGE and gels were blotted onto a nitrocellulose membrane. After blocking in Rotiblock (Carl Roth, Germany), first antibody was applied. Infrared-fluorescent-dye-conjugated secondary antibodies were purchased from Licor (Bad Homburg, Germany). Signals were detected with an infrared-based laser scanning detection system (Odyssey Classic, Licor, Bad Homburg, Germany). Uncropped scans are provided in the supplement (Supplementary Fig. 8).

### Oxygen consumption rate

The cellular OCR was analyzed using a Seahorse 96 extracellular flux analyzer (Agilent). HAEC were plated in Seahorse 96-well cell culture plates 1×10^4^ cells/well 1 day before the assay and equilibrated for 1 h in Krebs Henseleit buffer (111 mM NaCl, 4.7 mM KCl, 1.25 mM CaCl_2_, 2 mM MgSO_4_, 1.2 mM NaH_2_PO_4_) supplemented with 11 mM l-Glucose and 2 mM l-Glutamine. Cells were treated with 2.5 µM oligomycin A, 1 µM CCCP, 1 µg ml^−1^ antimycin A and 1 µM rotenone as indicated. OCR was normalized to protein content of the wells.

### Amino acid profiling by mass spectrometry

For amino acid profiling, HAEC were lysed in 85% Ultra LC-MS methanol (Roth, Cat# HN41.1). Lysates were centrifuged for 10 min at 17,000 × *g* and 50 µl of supernatants were processed using the EZ:faast LC-MS free amino acid analysis kit (Phenomenex, Cat# AL0-7500) according to the manufacturer’s instructions with minor modifications. An internal standard (10 µl) was applied to all samples and to the standard curve. The internal standards included homoarginine, methionine-D_3_ and homophenylalanine. Analysis of metabolites was performed by LC-MS/MS using an Agilent 1290 Infinity LC system (Agilent) coupled to a QTrap 5500 mass spectrometer (Sciex). The intensity of the measured metabolite was normalized to internal standards and protein content of cell lysate pellets. Analyst 1.6.2 and MultiQuant 3.0 (Sciex, Darmstadt, Germany) were used for data acquisition and analysis.

### HILIC-LC-MS/MS analysis of metabolomics flux

For serine heavy isotope tracking, HAEC were washed twice with glycine and serine-free EBM. HAEC were treated with or without oxPAPC in EBM without serine and supplemented with 300 µM ^13^C_3_-Serine (Cambridge Isotope Laboratories, #201595-68-8) and 1% dialyzed FBS (ThermoFisher Scientific, Cat# A3382001) for 24 h. For glycine heavy isotope tracking, HAEC were washed twice in glycine and serine free EBM. HAEC were treated with or without oxPAPC in EBM without glycine and supplemented with 30 µM 1,2-^13^C_2_-Glycine (Cambridge Isotope Laboratories, #67836-01-5) and 1% dialyzed FBS and for 24 h. Medium was harvested and centrifuged for 10 min at 17,000 × *g* and supernatant was subjected to mass spectrometry analysis. HAEC were lysed in 85% Ultra LC-MS methanol, centrifuged for 10 min at 17,000 × *g* and supernatant was subjected to mass spectrometry. Adenosine monophosphate (Sigma-Aldrich, Cat# A2002), NAD (Sigma-Aldrich, Cat# N1636), Inosine (Sigma-Aldrich, Cat# I4125), glycine (VWR, Cat# 10119CU), and serine (Sigma-Aldrich, Cat# S4500) were used as authentic reference standards. Liquid chromatography was performed using an Agilent 1290 Infinity LC system (Agilent, Waldbronn, Germany) coupled to a QTrap 5500 mass spectrometer (Sciex, Darmstadt, Germany). Electro spray ionization in positive and negative mode was employed, respectively. HILIC separation was achieved using a Nucleoshell HILIC column (Macherey-Nagel, Düren, Germany) 100 × 2 mm, 2.7 µm particle size, equipped with a HILIC guard column. The mobile phases were acetonitrile and 20 mM ammonium acetate (pH 9.1) at a flow rate of 450 µl min^−1^. Column temperature was set to 40 °C. The injection volume was 2.5 µl. The gradient started with 85% acetonitrile. Analyst 1.6.2 and MultiQuant 3.0 (Sciex, Darmstadt, Germany) were used for data acquisition and analysis, respectively.

### Nucleoside measurement by mass spectrometry

HAEC were lysed and centrifuged for amino acid profiling. Additionally, cell culture supernatant was collected and centrifuged for 10 min at 17,000 × *g*. 200 µl of samples were analyzed by liquid chromatography-tandem mass spectrometry (LC-MS/MS). The LC-MS/MS system consisted of an Agilent 1260 Series binary pump (Agilent Technologies) and a triple quadrupole mass spectrometer 5500 QTRAP (Sciex). Calibration was done using the following standard substances: Adenosine (Sigma-Aldrich, Cat# A9251), guanosine (Sigma-Aldrich, Cat# G6752), cytidine (Sigma-Aldrich, Cat# C4654), and uridine (Sigma-Aldrich, Cat# U3750). ^13^C_5_-adenosine (Alsachim, Cat# C2291), ^13^C_5_-guanosine (Alsachim, Cat# C2240), ^13^C_5_-cytidine (Omicron, Cat# NUC-056), and ^13^C_5_-uridine (Alsachim, Cat# M436) were used as internal standards. Analyst Software 1.6 (Sciex) was used for data acquisition and analysis. The intensity of measured nucleosides was normalized to internal standards and protein content of cell lysate pellets for intracellular nucleoside measurement and to intracellular RNA content for extracellular nucleoside measurement.

### ATP measurement

Extracellular ATP was measured in cell supernatant centrifuged for 3 min at 5000 × *g* and then 10 min at 17,000 × *g* using CellTiter-Glo Luminescent Cell Viability Assay (Promega, Cat# G7570). Luminescence was detected using an Infinite 200Pro plate reader (Tecan) and normalized to intracellular RNA concentration.

### Cell migration

A scratch wound-healing assay was performed in 24-well plates. HUVEC were scratched and cultured in EBM containing 1% FCS. Endothelial cell migration into the scratched area was monitored by live cell imaging (Zeiss TIRF System LASOS77). The distance migrated was calculated using ImageJ software.

### Spheroid outgrowth assay

HUVEC spheroids were generated as described^65^. Images were acquired with an Axiovert135 microscope (Zeiss). For quantification of the cumulative sprout number, ten spheroids per condition were analyzed with the help of the AxioVision software (Zeiss). Treatments with VEGF-A 165 were performed for 16 h with a concentration of 10 ng ml^−1^.

### Animal experiments

All animal experiments were performed in accordance with the National Institutes of Health Guidelines on the Use of Laboratory Animals. All animal procedures were approved by the local committees or regulatory authorities. Mouse experiments were carried out in the C57BL/6 strain and only male mice were used (Jackson Laboratory, Bar Harbor). Mice were fed ad libitum with standard chow diet (unless otherwise indicated) and experiments were carried out at an age of 8–12 weeks.

### Aortic ring assay

Aortic ring assay was conducted as the following: aortae from ten male wild-type 6–8-week-old mice were removed, cleaned, and embedded in a collagen type I gel (BD Biosciences, Heidelberg, Germany) in a 48-well plate containing EBM medium supplemented with murine orthologous serum (2%). For *Mthfd2* knockdown, scrambled control siRNA (Ambion) and *Mthfd2* siRNA (Sigma-Aldrich) in a final concentration of 100 µM were added in the growth media for 24 h by use of the Lipofectamine RNAi reagent (Invitrogen). Glycine treatment was performed in 500 µM final concentration every 48 h. Tube-like structures were allowed to develop over 5 days. Thereafter, the samples were fixed (4% paraformaldehyde) and endothelial cells were visualized using antibody against *Pcam1*. The knockdown was evaluated by immunostaining against *Mthfd2*.

### High-fat diet and oxPAPC ex vivo treatment

All animal procedures were approved by the local government authority Regierungspräsidium Darmstadt. For high-fat diet male *ApoE−/−* mice (C57BL/6 background) were purchased from Taconis M&B A/S (Ry, Denmark, strain B6.129P2-Apoetm1Unc N6) and bred at the local facility. Paigen-type (19.6% protein, 15.8% fat, 4.3% fiber, 5% ash, 24.4% starch, 7.9% sugar; 1.25% cholesterol, 0.5% Cholate) diet was purchased from Ssniff Spezialdiäten (Soest, Germany). Animals received paigen-type diet ad libitum for 1 or 4 days. After diet feeding, endothelial-specific RNA of carotid arteries were isolated^29^.

For ex vivo oxPAPC treatment C57BL/6J mice were euthanized with isoflurane. Mice were coated with 70% ethanol to decontaminate the incision area and then perfused with cold HANKS solution. The thoracic aorta was detached from the thoracic vertebrae and cleaned from adipose tissue. Vessels were cut into 1 mm segments and incubated in EBM 1% FCS (endothelial basal medium + 1% fetal calf serum). Each vessel was split into 12 × 1 mm segments, after which four segments were used for each treatment group. All vessels were incubated overnight in EBM + 1% FCS and treated the following day. RNA was isolated according to the manufacturer instructions (Bio&Sell RNA Isolation Kit).

### Mouse partial carotid ligation and RNA isolation and qRT-PCR

All animal procedures were approved by the Animal Care and Use Committee at Emory University. Mice were anesthetized with 3.5% isoflurane initially and then anesthesia was maintained with 1.5−2% during the entire procedure. Partial carotid ligation surgery was performed on the left carotid artery. Briefly, the LCA bifurcation was exposed by blunt dissection and three of four caudal LCA branches (left external carotid, internal carotid, and occipital arteries) were ligated with 6–0 silk sutures, leaving the superior thyroid artery patent. The contralateral RCA was left intact as an internal control. Mice were monitored post-surgery and were allowed to recover. Following surgery, analgesic buprenorphine (0.1 mg kg^−1^) was administrated. Post procedure, *d*-flow in the LCA of all animals was confirmed by ultrasonography to verify the success of partial ligation surgery.

After 48 h of partial carotid ligation surgery, endothelial-enriched RNA was collected from the carotids by flushing the lumen quickly with Qiazol. Briefly, LCA and RCA were quickly flushed with 150 μl of QIAzol lysis reagent (Qiagen) using 29G insulin syringe into a microfuge tube. The eluate was then used for total intimal RNA isolation using the miRNeasy mini kit (Qiagen) following the manufacturer’s protocol.

Total RNA was polyadenylated and reverse transcribed for use in a two-step qPCR setup using High-capacity cDNA synthesis kit (ABI) and using Brilliant II SYBR Green QPCR Master Mix (Stratagene) with custom-designed primers on a Real-Time PCR System (StepOne Plus, Applied Biosystems). Fold changes between endothelial enriched RNAs from LCA and RCA were determined for all target genes using the ∆∆Ct method. Initial quality control for endothelial-enrichment of the intimal RNA extracted was determined by qPCR for endothelial markers gene (*Pcam1*) and non-endothelial marker gene, *Acta2* (smooth muscle cell marker) and *Cd11b* (leukocyte marker gene). We also tested the expression of well-known flow-sensitive genes, such as *Klf2* and *Klf4*. All samples that passed our initial quality control check were used for subsequent qPCR analysis.

### Zebrafish experiments

All experimental procedures on animals were approved by the local government authority Regierungspräsidium Karlsruhe (license no.: 35-9185.64) and carried out in accordance with the approved guidelines. Embryos of the *Tg*(*fli1:EGFP*)*y1* line were raised and staged while kept in E3 medium (5 mM NaCl, 0.17 mM KCl, 0.33 mM CaCl_2_, 5–10% methylene blue) at 28.5 °C with or without 0.003% 1-phenyl-2-thiourea (Sigma) to suppress pigmentation and staged according to somite number or hours post-fertilization (hpf). The experimental groups were not blinded and all relevant data are available from the authors.

Morpholinos (Gene Tools) were diluted in 0.1 M KCl and injected through the chorion of one-cell or two-cell stage embryos. Morpholino *mhfd2* (5′-TGTAATAGAGAGTGTCTTACCAACT-3′) or standard control morpholino (5′- CCTCTTACCTCAGTTACAATTTATA -3′) were used and dose escalation studies were performed to determine submaximal morpholino concentrations. Knockdown was confirmed by RT-PCR with the primers 5′-GCGATTTAGCCGTTTGTGAG-3′ and 5′-TCAAGATGGTCTCGCTGCTG-3′ for *mthfd2* and 5′-ACGGTCAGGTCATCACCATC-3′ and 5′-TGGATACCGCAAGATTCCAT-3′ for β-actin. PCR was conducted at 95 °C for 3 min (95 °C for 30 s, 60 °C for 30 s, 72 °C for 30 s)×32 cycles and 72 °C for 5 min and PCR products were loaded on an agarose gel. OxPAPC was diluted in 0.1% BSA/PBS and injected in the one-cell stage^66^. *Tg*(*fli1:EGFP*)*y1* embryos were manually dechorionated and anesthetized with 0.05% tricaine (Sigma). Morphological analysis of vessels was performed using a CTR 6000 microscope (Leica) or a TCS SP5 system (Leica). For quantitative morphological analysis the number of intact and partial normal intersegmental vessels (ISV) and dorsal longitudinal anastomotic vessel (DLAV) connections between the investigated ISVs in the trunk vasculature was determined at 72 hpf. Seventeen consecutive ISVs and 16 DLAV connections per embryo were analyzed for the anterior part between head and anus starting at the sixth ISV. The number of embryos per experimental conditions was used as previously described^67^. Results are given as mean number of vessels per embryo ± SE. For RT-qPCR analysis of *mthfd2*, 4–5 zebrafish embryos were pooled per sample and homogenized in RNA-Lysis buffer (Bio&Sell) using a canula. RNA isolation and RT-qPCR was conducted as described above for HAEC.

### HDL isolation

Samples were collected according to the study approved by the ethics committee in Zurich (Kantonale Ethik-Kommission Zürich (KEK), Switzerland, KEK-ZH-Nr. 2012-0321). HDL was separated from LDL and albumin via two ultracentrifugation steps using a potassium bromide (KBr) density solution. In particular, 500 µL serum from age-matched healthy subjects and stable CAD patients were centrifuged for 6 h after adding 500 µl KBr (*d* = 1.245 g cm^−3^) and 1 ml 0.150 M sodium chloride in 1 mM EDA solution. After removal of LDL the remaining HDL was added to KBr (*d* = 1.32 g cm^−3^) in order to reach a final density of 1.21 g cm^−3^ and was then centrifuged for 16 h at 4 °C (full protocol available on demand). The isolated HDL was purified by dialysis with 0.9% NaCl and 30 K Amicon Ultra Centrifugal Filters (Millipore). Dialyzed HDL samples were resuspended in 300 µl of 0.9% NaCl and protein concentration was determined using Pierce BCA Protein Kit. Samples were overlaid with Argon after isolation and stored no longer than 2 weeks at 4 °C.

### Atherosclerosis human cohort experiments

The present study followed the Code of Ethics of the World Medical Association (Declaration of Helsinki) and was approved by the Scientific and Ethic Committee of Hipokrateion University Hospital (PN1539/addition N.2822/2018) and all patients enrolled gave their informed consensus. Carotid plaques were prospectively collected from 40 random patients, who had internal carotid artery stenosis of 80–90% and underwent carotid endarterectomy (Hippocration Hospital, Athens, Greece). Extent demographic and clinical data are presented in Supplementary Table 7. Stenotic lesions were divided into two groups, namely stable plaques (stable), and unstable plaques (unstable). Twenty additional samples from healthy thyroid arteries were used as the control group.

Plasma from 60 patients, 26 characterized with stable atherosclerotic plaques, 26 patients characterized with unstable atherosclerotic plaques and 20 healthy donors was used for amino acid profiling by mass spectrometry as described above. Plasma samples were collected from the cohort used for the qRT-PCR and western blot analysis with addition of six patients characterized as asymptomatic/stable, six patients characterized as symptomatic/unstable and additional 12 healthy subjects.

For RT-qPCR total RNA was extracted using an RNeasy kit (QIAGEN, Hilden, Germany), and equal amounts (1 µg) of total RNA was reverse transcribed (Superscript III; Invitrogen). Gene expression levels were detected using SYBR Green (Absolute QPCR SYBR Green Mix; Thermo Fisher Scientific).

For western blot analysis samples were lysed in ice-cold RIPA buffer (50 mmol l^−1^ Tris HCl (pH 7.5), 150 mmol l^−1^ NaCl, 25 mmol l^−1^ NaF,10 mmol l^−1^ Na_4_P_2_O_7_,1% Triton X-100 and 0.5% sodium deoxycholate) supplemented with 0.1% SDS as well as protease and phosphatase inhibitors. Proteins were then precipitated in acetone (4/1 v v^−1^) overnight at −80 °C. Following centrifugation (16,000 × *g*, 15 min, 4 °C) the pellets were resuspended in ice-cold RIPA buffer containing 1% SDS and protease and phosphatase inhibitors. Samples (1 mg ml^−1^) were passed through desalting columns (Thermo Scientific) and the proteins recovered were used for subsequent evaluation.

### Statistics for wet-laboratory experiments

For wet-laboratory experiments, a group size of *n* = 6 was chosen for most of the cell functional assays due to their larger variability and on the basis of previous experiences. For more accurate methods or for supportive data or when similar experiments were carried out in different subgroups samples sizes for *n* = 3 to 4 were considered sufficient. A total of four different batches of HUVEC was used throughout the study. Unless otherwise indicated, data are given as mean ± standard error of the mean. Calculations were performed with Prism 5.0 or BiAS.10.12. The latter was also used to test for normal distribution and similarity of variance. In the case of multiple testing, Bonferroni correction was applied. For multiple group comparisons, analysis of variance followed by post-hoc testing was performed (ANOVA with Bonferroni for normally distributed data and ANOVA with Newman-Keuls test for non-normal distributions). Individual statistics of dependent samples were performed by paired *t* test, of unpaired samples by unpaired *t* test, and, if not normally distributed, by Mann–Whitney test. *p* values of <0.05 were considered as significant. Unless otherwise indicated, *n* indicates the number of individual experiments. Calculations of cohort size for animal experiments were based on an alpha-error of 0.05, beta of 0.2 and a relative difference of 0.3. According to RRR principles, experiments were terminated either when significance was reached or when there was obviously no effect. Assignment to treatment groups in animal experiments was based on random selection of the animals. Sham and intervention studies were also performed side by side and not consecutively. Blinding was not performed.

### Data availability

The validation data set (RNA sequencing of siRNA control and siRNA *MTHFD2* in HAEC) has been deposited in GEO and is accessible with the accession code GSE100261 at (https://www.ncbi.nlm.nih.gov/geo/). The Bayesian control and oxPAPC networks are available in the supplement (Supplementary Data 1,2). The Bayesian network reconstruction methods are implemented in the software suite RIMBANET (Reconstructing Integrative Molecular BAyesian NETworks)^68^ which is available at (http://research.mssm.edu/integrative-network-biology/RIMBANET/RIMBANET_overview.html). The gene expression data of HAECs are described in Romanoski et al.^5^ and is available as the GEO data set GSE30169. The expression data were adjusted for batch effect before inputting into differential connectivity analysis and Bayesian network constructions. The HAECs SNP genotype data are described in Romanoski et al.^69^. The genotype data are available upon request.

## Electronic supplementary material

Supplementary Information

Description of Additional Supplementary Files

Supplementary Data 1

Supplementary Data 2

Supplementary Data 3

Supplementary Data 4

Supplementary Data 5
