# Supplementary material for: Oxidized phospholipids regulate amino acid metabolism through MTHFD2 to facilitate nucleotide release in endothelial cells
Source: Nat Commun. 2018 Jun 12;9:2292. doi: 10.1038/s41467-018-04602-0 (PMC5997752; doi:10.1038/s41467-018-04602-0)
Supplement: Supplementary file 1 — Supplementary Information [file 41467_2018_4602_MOESM1_ESM.pdf]

# Oxidized phospholipids regulate amino acid metabolism through MTHFD2 to facilitate nucleotide release in endothelial cells

**Hitzel et al.**

## Supplementary Figures

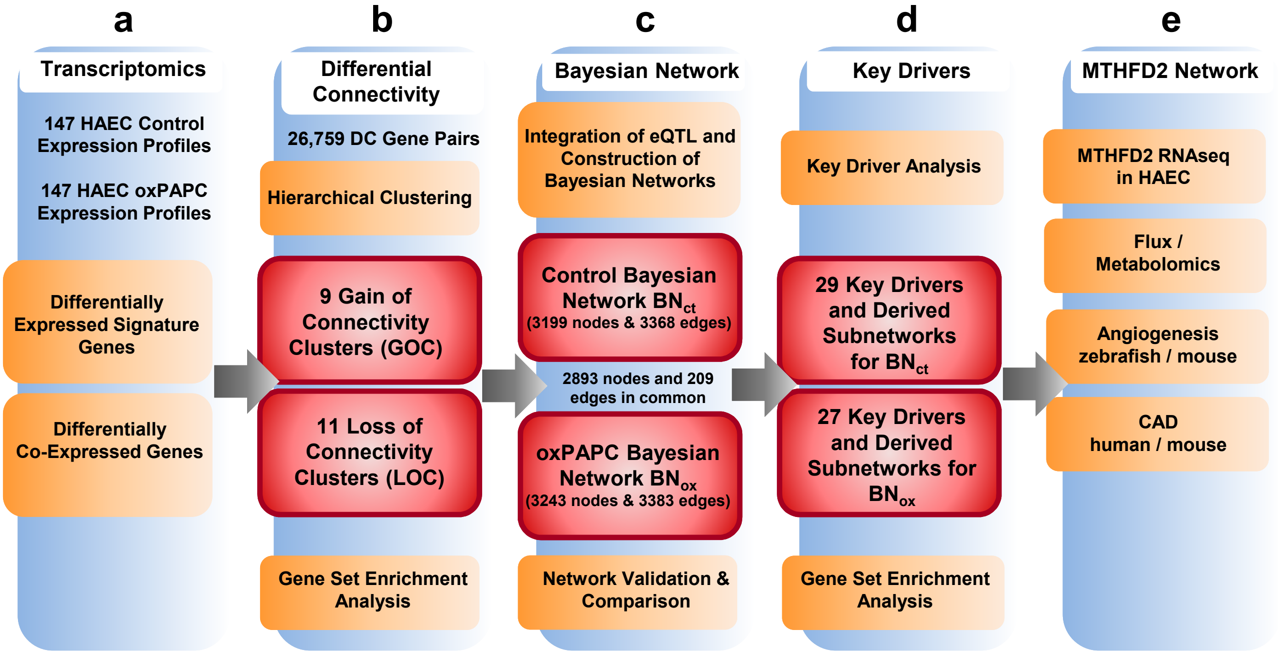


**Supplementary Figure 1 Integrative network approach in HAEC.** Expression profiles (a) were used to compute differential connectivity clusters (b) and genotype profiles were integrated to construct Bayesian networks (c). Key drivers of the Bayesian networks were identified (d) and the subnetwork of the key driver MTHFD2 was investigated in detail (e).

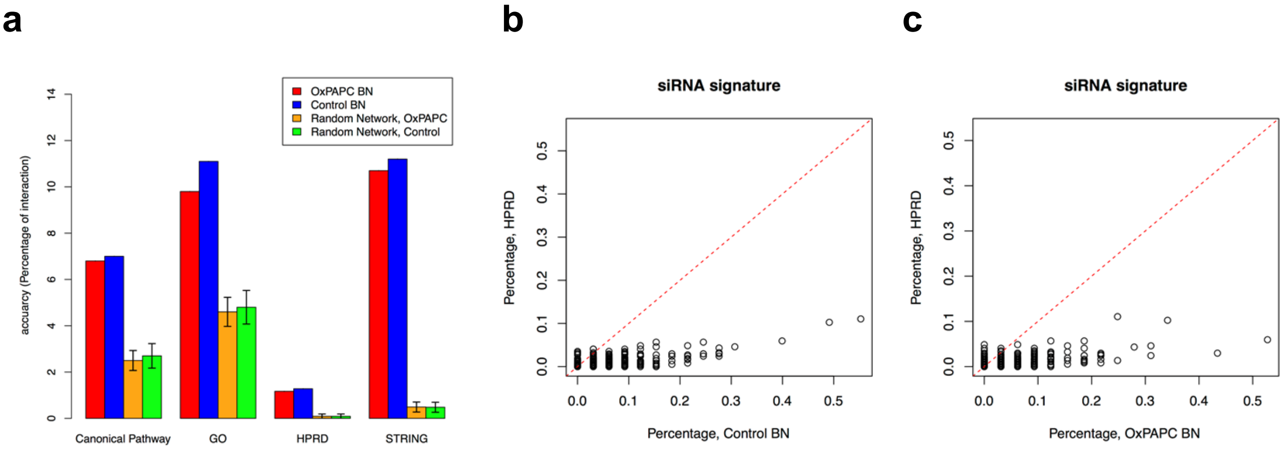


**Supplementary Figure 2 Assessment of HAEC Bayesian networks. (a)** Estimated accuracy of Bayesian networks (BN) based on four independent databases of gene networks and gene sets, including canonical pathway, Gene ontology (GO), Human Protein Reference Database (HPRD), and Search Tool for the Retrieval of Interacting Genes/Proteins database (STRING). The percentage of inferred gene-gene connections that are in existing protein/gene network databases is shown. For the random networks, the mean of accuracy is shown based on 100 random networks. For all the reference databases considered, the estimated accuracy of our Bayesian networks is significantly greater than random networks. **(b), (c)** Comparison of estimated accuracies for constructed Bayesian networks and HPRD networks. For each gene set that is significantly regulated by siRNA treatment in human umbilical vein endothelial cells, the percentage of inferred gene-gene connections that are within each gene set is shown based on our constructed control Bayesian network (x-axis) (b) or oxPAPC Bayesian network (x-axis) (c) and HPRD (y-axis).

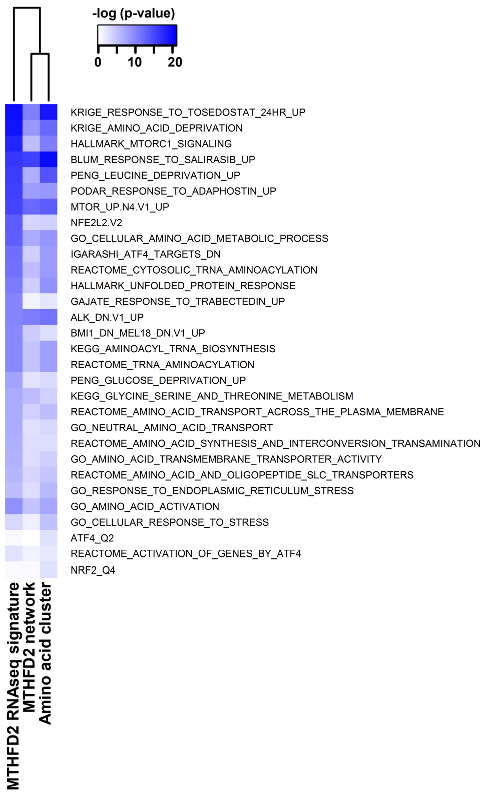


**Supplementary Figure 3 Canonical and non-canonical gene set enrichment.** Heatmap of P-values from selected canonical and non-canonical gene set categories (MSigDB) for *MTHFD2* RNAseq signature (FDR<0.05), amino acid cluster and *MTHFD2* network. *NFE2L2* = Nrf2 (Nuclear Factor, Erythroid 2 Like 2), *BMI1* = BMI1 Proto-Oncogene, Polycomb Ring Finger.

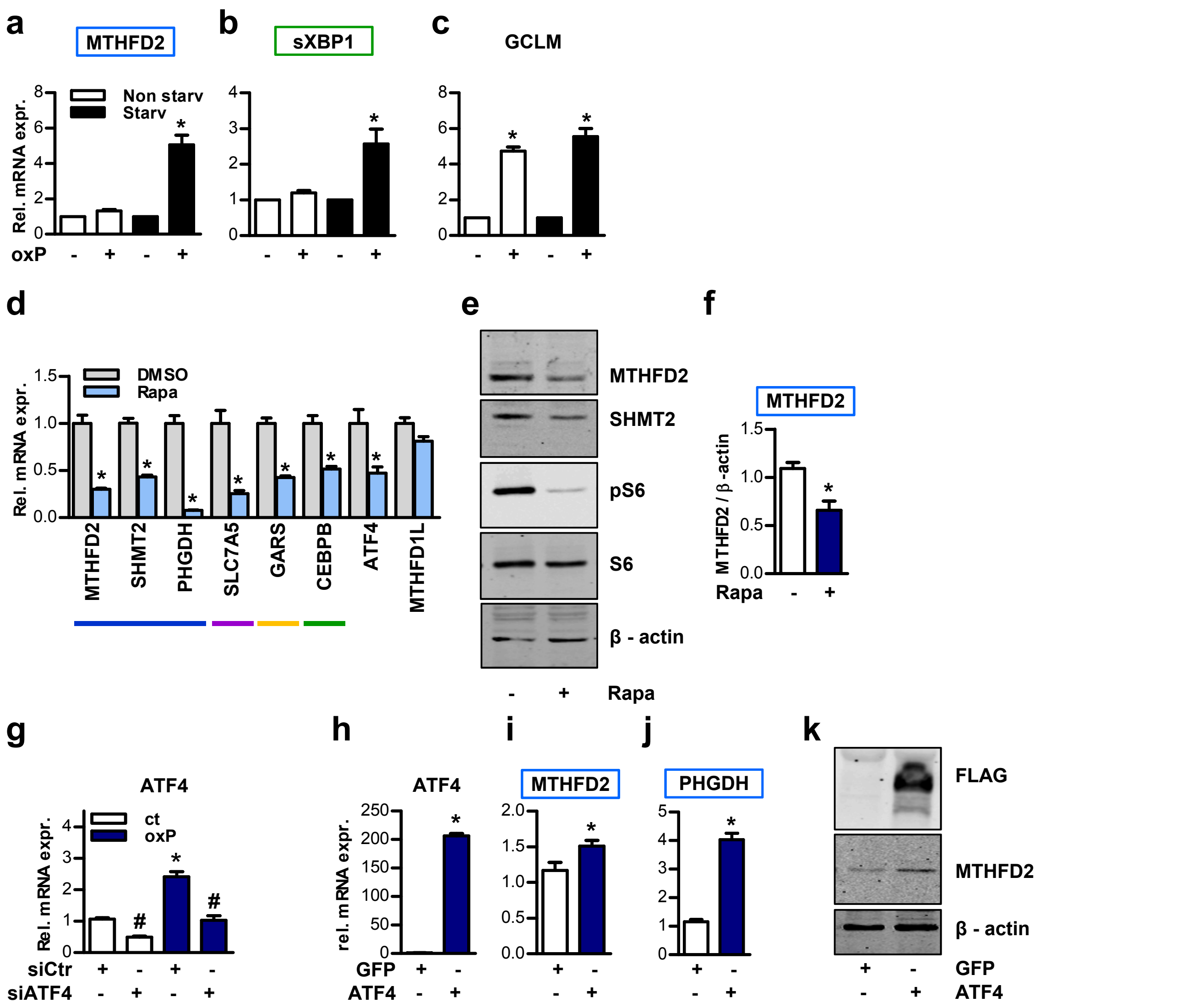


**Supplementary Figure 4 Regulatory pathways contributing to oxPAPC induced *MTHFD2* network expression. (a)-(c)** Quantitative RT-PCR detection. HAEC were exposed to growth medium (8% FCS) (Non starv) with or without oxPAPC (oxP) or basal medium (1% FCS) (Starv) with or without oxPAPC (n=4). Genes belonging to *MTHFD2* network are framed as in Fig. 3a. *sXBP1* = spliced X-Box Binding Protein 1, *GCLM* = Glutamate-Cysteine Ligase Modifier Subunit. **(d)** Relative mRNA expression of genes belonging to the *MTHFD2* network as well as *ATF4* and *MTHFD1L* in HAEC exposed to rapamycin in growth medium (Rapa, 20 nM) or DMSO for 16 h (n=12). Genes belonging to *MTHFD2* network are highlighted as in Fig. 3a. **(e),(f)** Western Blot detection (e) and quantification (f) of HAEC treated with rapamycin in growth medium (20 nM) or DMSO for 16 h (n=4). (*P ≤ 0.05, Student's t-test) **(g)** qRT-PCR detection of *ATF4* in HAEC treated as in Fig. 4 (j). **(h)-(j)** Relative mRNA expression in HAEC overexpressing GFP or Flag-tagged *ATF4* for 24 h (n=4). (*P ≤ 0.05, Student's t-test) **(k)** Western Blot analysis of *MTHFD2* in HAEC overexpressing FLAG-tagged *ATF4* or GFP empty vector as control for 24 h. Data are represented as mean ± SEM, *P ≤ 0.05, #P ≤ 0.05 (*ATF4* vs Control siRNA) (ANOVA with Bonferroni post-hoc test if not otherwise indicated).

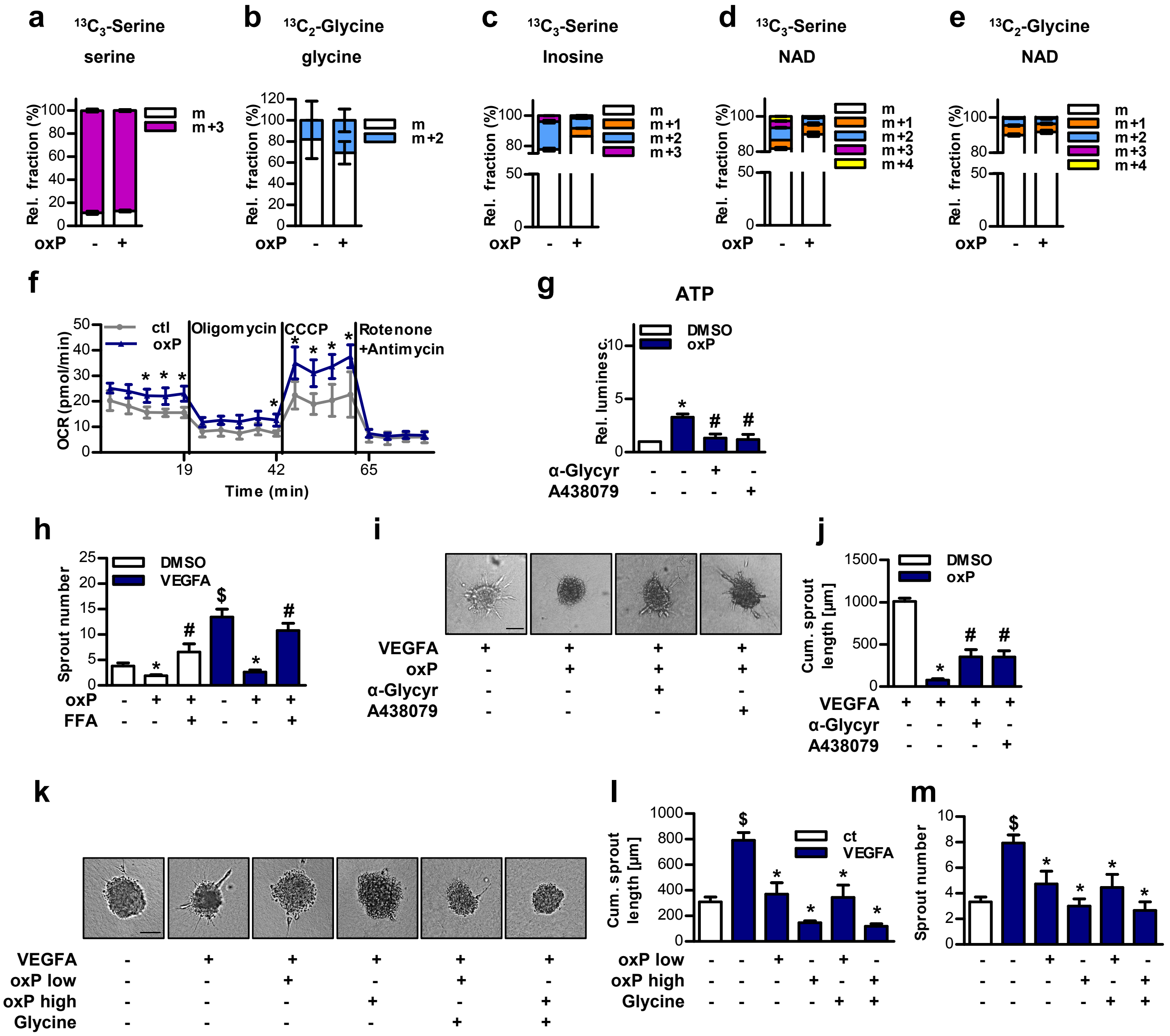


**Supplementary Figure 5 VNUT inhibitors rescue normal sprouting after oxPAPC treatment. (a),(b)** HAEC were treated with $^{13}C_3$-serine (a) or $^{13}C_2$-glycine (b) and oxPAPC (oxP) or control for 24 h and cells were lysed and measured by mass spectrometry (n=3). Relative fractions of intracellular non-heavy serine (m) or imported heavy-serine (m+3) (a) and relative intracellular fractions of non-heavy glycine (m) and imported heavy-glycine (m+2) (b) are shown. **(c)-(e)** HAEC were treated with $^{13}C_3$-serine (c),(d) or $^{13}C_2$-glycine (e) and oxPAPC or control for 24 h and supernatants were measured by mass spectrometry (n=3). Relative fractions of extracellular purine derivatives inosine (c) and NAD (d),(e) containing no (m), one (m+1), two (m+2), three (m+3) or four (m+4) heavy carbons are shown. **(f)** Oxygen consumption rate (OCR) profile as an index of mitochondrial respiration in HAEC exposed for 4 h to medium (1% FCS) with or without oxPAPC (n≥3). HAEC were treated with the ATP synthase inhibitor Oligomycin (2.5 µM), with CCCP (1 µM) for maximal mitochondrial capacity and with antimycin A (1 µg/ml) and rotenone (1 µM) to inhibit mitochondrial activity. (ANOVA with repeated measures) **(g)** ATP measurement of supernatants of HAEC exposed to medium (1% FCS) with or without oxPAPC and α-glycyrrhetinic acid (50 µM) or A438079 (100 µM) for 8 hours. ATP was measured by luminescence and was normalized to the intracellular RNA concentration (n≥3). **(h)** Sprout number in the spheroid outgrowth assay in Fig. 5p of human umbilical vein endothelial cells treated with combinations of oxPAPC, flufenamic acid (FFA, 50 µM) and VEGF-A165 (10 ng $ml^{-1}$) as indicated (n=6). **(i),(j)** Spheroid assay (i) and quantification (j) of the cumulative sprout length of human umbilical vein endothelial cells treated with oxPAPC, α-glycyrrhetinic acid (50 µM) or A438079 (100 µM) as indicated. All samples were treated with VEGF-A165 (10 ng $m^{-1}l$) (n=3). Scale bar: 50 µM. **(k)-(m)** Spheroid assay (k) and quantification of the cumulative sprout length (l) and sprout number (m) of human umbilical vein endothelial cells treated with low oxPAPC concentration, high oxPAPC concentration, glycine (500 µM) and VEGF-A165 (10 ng/ml) as indicated (n=6). Scale bar: 50 µM. Data are represented as mean ± SEM, *P ≤ 0.05 (oxP vs Ct), $^{\#}$P ≤ 0.05 (inhibitor present vs absent), $^{\$}$P ≤ 0.05 (VEGFA vs Ct), (ANOVA with Bonferroni post-hoc test if not otherwise indicated).

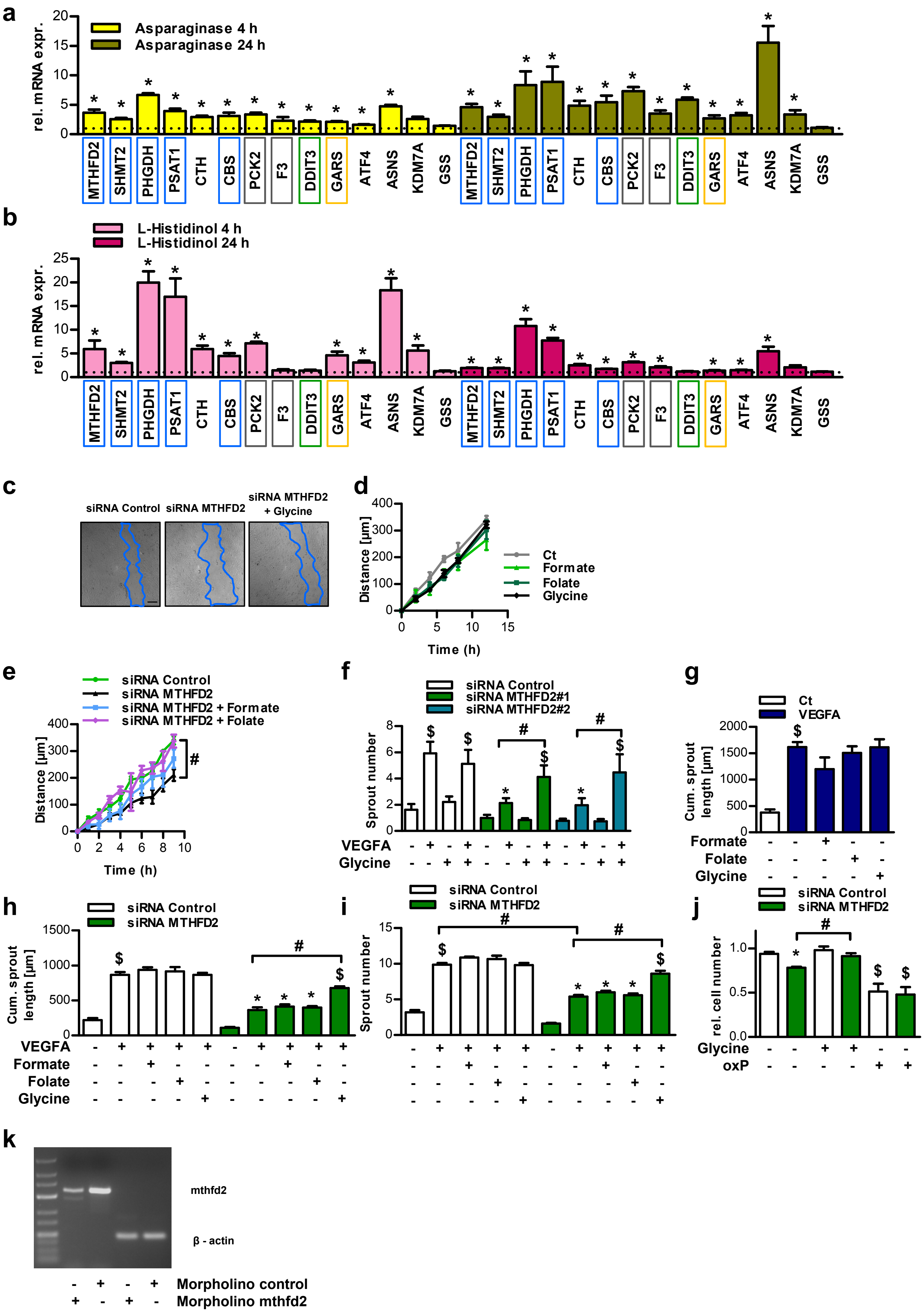

a
Asparaginase 4 h
Asparaginase 24 h
rel. mRNA expr.
MTHFD2
SHMT2
PHGDH
PSAT1
CTH
CBS
PCK2
F3
DDIT3
GARS
ATF4
ASNS
KDM7A
GSS
b
L-Histidinol 4 h
L-Histidinol 24 h
c
siRNA Control
siRNA MTHFD2
siRNA MTHFD2 + Glycine
d
Distance [µm]
Time (h)
Ct
Formate
Folate
Glycine
e
siRNA Control
siRNA MTHFD2
siRNA MTHFD2 + Formate
siRNA MTHFD2 + Folate
f
siRNA Control
siRNA MTHFD2#1
siRNA MTHFD2#2
Sprout number
VEGFA
Glycine
g
Ct
VEGFA
Cum. sprout length [µm]
Formate
Folate
Glycine
h
siRNA Control
siRNA MTHFD2
Cum. sprout length [µm]
VEGFA
Formate
Folate
Glycine
i
siRNA Control
siRNA MTHFD2
Sprout number
j
siRNA Control
siRNA MTHFD2
rel. cell number
Glycine
oxP
k
mthfd2
β - actin
Morpholino control
Morpholino mthfd2

**Supplementary Figure 6 Impact of amino acid and folate metabolism on endothelial cells. (a)** Quantitative RT-PCR detection. HAEC were treated with 1 U $ml^{-1}$ Asparaginase (ASNase) or $H_2O$ in growth medium for 4 and 24 hours (n≥4). Genes belonging to *MTHFD2* network are framed as in Fig. 3a. *CTH* = cystathionine gamma-lyase, *CBS* = cystathionine-beta-synthase, *F3* = Coagulation factor III / tissue factor, *ASNS* = asparagine synthetase, *KDM7A* = Lysine Demethylase 7A, GSS = Glutathione Synthetase. **(b)** Quantitative RT-PCR detection. HAEC were treated with L-Histidinol (2 µM) or $H_2O$ in growth medium for 4 and 24 hours (n≥4). Genes belonging to *MTHFD2* network are framed as in Fig. 3a. **(c)** Representative image of migration scratch wound assay in Fig. 6g. Scale bar: 100 µM. **(d)** Migration distance in a scratch wound assay of human umbilical vein endothelial cells treated with formate (50 µM), folate (10 µM) or glycine (500 µM) for the indicated time points (n=3). **(e)** Migration as in (d) of human umbilical vein endothelial cells treated with siRNA against scramble control or *MTHFD2* (n=3). (Anova with repeated measures) **(f)** Quantification of sprout number of spheroid assay in Fig. 6h (n=6). **(g)** Quantification of the cumulative sprout length of a spheroid assay in human umbilical vein endothelial cells treated with formate (50 µM), folate (10 µM), glycine (500 µM) and VEGF-A165 (10 ng $ml^{-1}$) as indicated (n=8). **(h),(i)** Quantification of cumulative sprout length (h) and sprout number (i) of spheroid assay as in (g). Human umbilical vein endothelial cells were additionally treated with siRNA against scramble control or *MTHFD2* (n=3). **(j)** Relative cell number of human umbilical vein endothelial cells treated with glycine (500 µM) or oxPAPC for 24h. Cells were counted 48 h after transfection with scramble control or *MTHFD2* siRNA (n≥6). **(k)** RT-PCR for mthfd2 and β-actin in *tg(fli1:EGFP)* embryos at 72 hours post-fertilization injected with control or mthfd2 morpholino at 0.5 hours post-fertilization. One single experiment is shown. Data are represented as mean ± SEM, *P ≤ 0.05 (*MTHFD2* vs Control siRNA), $^{\#}$P ≤ 0.05 (with vs without amino acid or folate), $^{\$}$P ≤ 0.05 (VEGFA or oxP vs Ct) (ANOVA with Bonferroni post-hoc test).

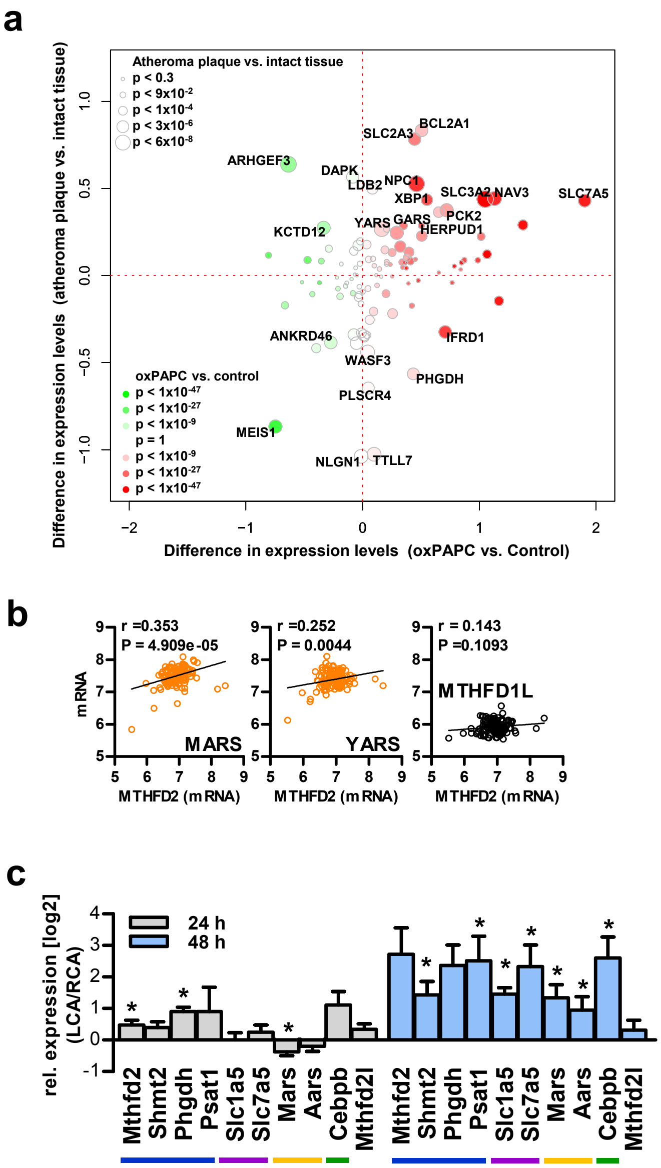


**Supplementary Figure 7 Expression of *MTHFD2* network in cardiovascular disease models. (a)** Expression of genes in *MTHFD2* network in human aortic plaques. Difference in expression levels of genes in *MTHFD2* network in 32 human atheroma plaques compared to 147 oxPAPC treated HAEC. **(b)** Scatter plots (additional to Fig. 7b) showing expression correlation in 126 human carotid plaque samples between *MTHFD2* and the *MTHFD2* network genes *MARS* and *YARS* (colored according to Fig 3a) as calculated by Pearson correlation. There was no correlation with the non-*MTHFD2* network gene *MTHFD1L* **(c)** Log2 expression profiles of representative genes within the *MTHFD2* network in the endothelium of healthy right carotid artery (RCA) compared to partially ligated left carotid artery (LCA) 24 h and 48 h weeks post ligation (n=5). Genes belonging to *MTHFD2* network are highlighted as in Fig. 3a. Data are represented as mean ± SEM, *$P \leq 0.05$ (ANOVA with Bonferroni post-hoc test).

## Fig. 4h

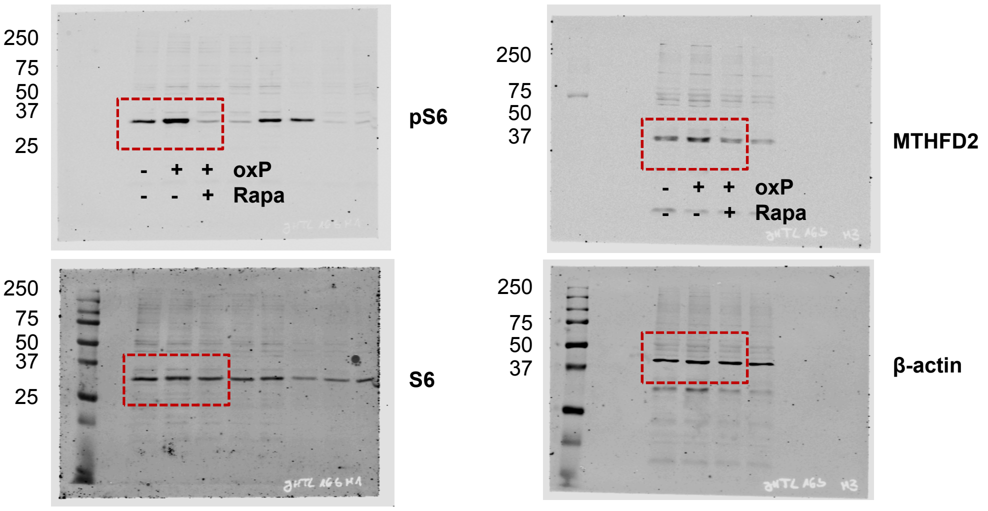


## Fig. 7e

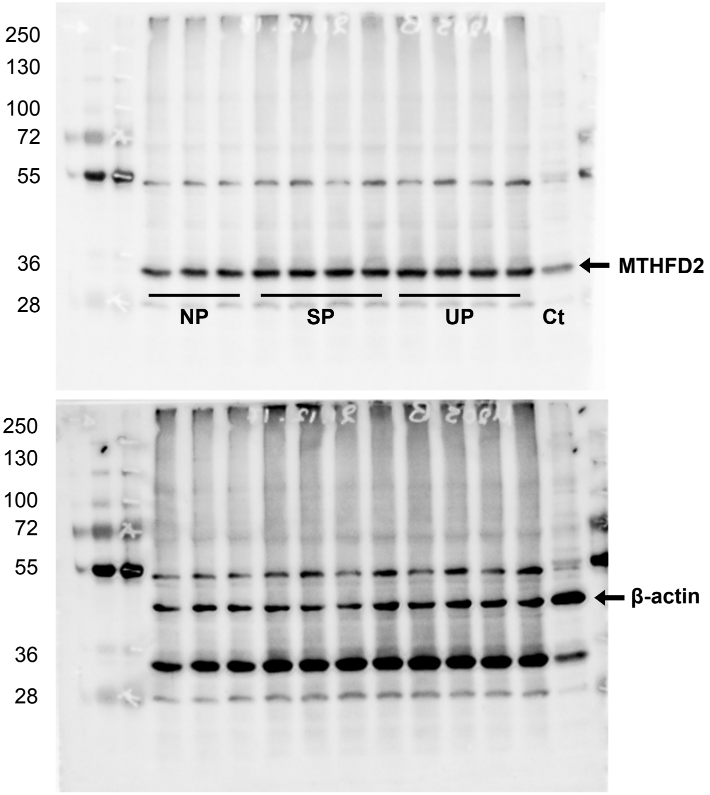


## Fig. 7r

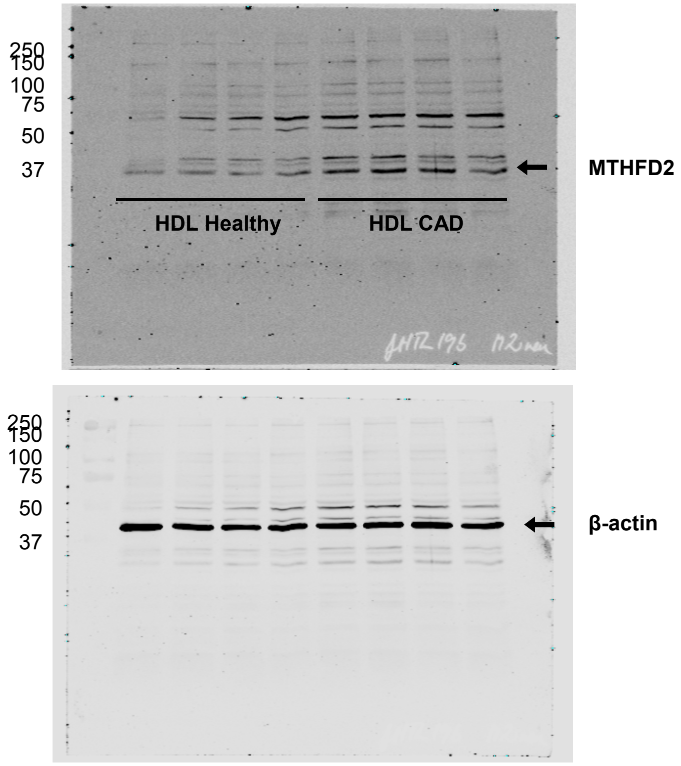


**Supplementary Figure 8 Full scans of Western Blots.**

## Supplementary Tables

**Supplementary Table 1 Canonical pathway enrichment in gain of connectivity clusters.**

| GOC cluster | Top functional category | p–value |
|---|---|---|
| GOC1 | GO_MACROAUTOPHAGY | 2.55E-03 |
| GOC2 | GO_HOMOPHILIC_CELL_ADHESION_VIA_PLASMA_MEMBRANE_ADHESION_MOLECULES | 3.22E-34 |
| | KEGG_LYSOSOME | 2.21E-10 |
| GOC3 | GO_LAMELLIPODIUM | 1.81E-05 |
| GOC4 | GO_MITOTIC_CELL_CYCLE | 2.12E-31 |
| | HALLMARK_G2M_CHECKPOINT | 1.11E-21 |
| | REACTOME_CELL_CYCLE | 4.35E-19 |
| | KEGG_CELL_CYCLE | 2.06E-05 |
| GOC5 | GO_NEGATIVE_REGULATION_OF_MITOTIC_CELL_CYCLE | 7.74E-05 |
| GOC6 | GO_CELLULAR_AMINO_ACID_METABOLIC_PROCESS | 1.63E-09 |
| | HALLMARK_MTORC1_SIGNALING | 2.66E-11 |
| | REACTOME_CYTOSOLIC_TRNA_AMINOACYLATION | 4.24E-09 |
| | KEGG_AMINOACYL_TRNA_BIOSYNTHESIS | 1.19E-08 |
| GOC7 | GO_ORGAN_MORPHOGENESIS | 8.13E-08 |
| GOC8 | GO_NUCLEIC_ACID_BINDING_TRANSCRIPTION_FACTOR_ACTIVITY | 6.28E-08 |
| GOC9 | GO_REGULATION_OF_CELLULAR_RESPONSE_TO_HEAT | 6.33E-10 |

The top functional GO category and the top functional categories from KEGG, HALLMARK and REACTOME gene sets (if *P*-value ≤ 1E-05) of each gain of connectivity (GOC) cluster are listed. Clusters are colored according to Fig. 1a.

**Supplementary Table 2 Canonical pathway enrichment in loss of connectivity clusters.**

| LOC cluster | Top functional category | p–value |
|---|---|---|
| LOC1 | GO_REGULATION_OF_MESODERM_DEVELOPMENT | 2.91E-05 |
| | HALLMARK_ESTROGEN_RESPONSE_LATE | 5.49E-05 |
| LOC2 | GO_POSTTRANSCRIPTIONAL_REGULATION_OF_GENE_EXPRESSION | 1.96E-06 |
| | HALLMARK_P53_PATHWAY | 3.66E-07 |
| LOC3 | GO_CATABOLIC_PROCESS | 6.59E-07 |
| | REACTOME_METABOLISM_OF_RNA | 9.46E-05 |
| LOC4 | GO_DNA_DEPENDENT_DNA_REPLICATION | 2.49E-09 |
| | REACTOME_CELL_CYCLE | 4.18E-09 |
| | HALLMARK_E2F_TARGETS | 1.17E-08 |
| | KEGG_DNA_REPLICATION | 7.28E-05 |
| LOC5 | GO_MITOCHONDRIAL_PART | 2.01E-10 |
| | HALLMARK_MYC_TARGETS_V1 | 5.02E-10 |
| | REACTOME_CELL_CYCLE | 4.29E-07 |
| LOC6 | GO_SPHINGOLIPID_METABOLIC_PROCESS | 1.09E-04 |
| LOC7 | GO_REGULATION_OF_CELLULAR_AMIDE_METABOLIC_PROCESS | 1.81E-06 |
| | HALLMARK_TNFA_SIGNALING_VIA_NFKB | 3.01E-06 |
| LOC8 | GO_RIBONUCLEOTIDE_BINDING | 2.39E-09 |
| | HALLMARK_G2M_CHECKPOINT | 9.23E-11 |
| LOC9 | GO_NCRNA_PROCESSING | 2.65E-09 |
| | REACTOME_SRP_DEPENDENT_COTRANSLATIONAL_PROTEIN_TARGETING_TO_M | 3.61E-08 |
| | EMBRANE | 9.28E-07 |
| | KEGG_RIBOSOME | |
| LOC10 | GO_IMMUNE_SYSTEM_PROCESS | 1.40E-07 |
| | KEGG_CELL_ADHESION_MOLECULES_CAMS | 1.41E-06 |
| | REACTOME_IMMUNOREGULATORY_INTERACTIONS_BETWEEN_A_LYMPHOID_AN | 8.34E-05 |
| | D_A_NON_LYMPHOID_CELL | |
| LOC11 | GO_CIRCULATORY_SYSTEM_DEVELOPMENT | 3.90E-12 |
| | HALLMARK_TGF_BETA_SIGNALING | 1.18E-06 |
| | KEGG_TGF_BETA_SIGNALING_PATHWAY | 2.17E-06 |

The top functional GO category and the top functional categories from KEGG, HALLMARK and REACTOME gene sets (if *P*-value ≤ 1E-05) of each loss of connectivity (LOC) cluster are listed. Clusters are colored according to Fig. 1b.

**Supplementary Table 3 Canonical pathway enrichment in amino acid cluster.**

| Amino acid cluster: Top functional canonical gene set categories | p–value |
|---|---|
| **MTOR_UP.N4.V1_UP** | 4.74E-14 |
| **ALK_DN.V1_UP** | 3.24E-12 |
| **HALLMARK_MTORC1_SIGNALING** | 2.66E-11 |
| **HALLMARK_UNFOLDED_PROTEIN_RESPONSE** | 1.07E-09 |
| **GO_CELLULAR_AMINO_ACID_METABOLIC_PROCESS** | 1.63E-09 |
| **REACTOME_CYTOSOLIC_TRNA_AMINOACYLATION** | 4.24E-09 |
| **GO_ORGANIC_ACID_METABOLIC_PROCESS** | 8.75E-09 |
| **KEGG_AMINOACYL_TRNA_BIOSYNTHESIS** | 1.19E-08 |
| **REACTOME_TRNA_AMINOACYLATION** | 1.19E-08 |
| **TGANTCA_AP1_C** | 3.83E-08 |
| **RCGCANGCGY_NRF1_Q6** | 4.61E-08 |
| **GO_NEGATIVE_REGULATION_OF_CELL_DEATH** | 5.55E-08 |

The top 12 canonical gene sets and *P*-value are shown. *MTOR* = mechanistic Target of Rapamycin, *ALK* = Anaplastic Lymphoma Kinase, *AP1* = Activator protein 1, *NRF1* = Nuclear Respiratory Factor 1.

**Supplementary Table 4 Extended list of key driver subnetworks in control Bayesian network (Fig. 2a).**

| KD | size | Top functional canonical gene set category | p–value |
|---|---|---|---|
| CDCA3 | 589 | GO_DNA_REPLICATION | 6.58E-19 |
| PDSS1 | 544 | GO_RIBOSOME_BIOGENESIS | 5.83E-10 |
| DOCK9 | 348 | GO_INORGANIC_ION_TRANSMEMBRANE_TRANSPORT | 5.85E-06 |
| NEK2 | 317 | HALLMARK_E2F_TARGETS | 5.28E-24 |
| TRAM1 | 281 | KEGG_WNT_SIGNALING_PATHWAY | 4.73E-05 |
| TBC1D8 | 268 | GO_INORGANIC_ION_TRANSMEMBRANE_TRANSPORT | 8.52E-05 |
| EMG1 | 243 | GO_TRNA_PROCESSING | 1.13E-04 |
| CKS1B | 232 | GO_DNA_REPLICATION | 7.56E-29 |
| ITGAV | 230 | HALLMARK_INFLAMMATORY_RESPONSE | 1.70E-06 |
| PBK | 223 | GO_DNA_REPLICATION | 2.86E-19 |
| UBXN4 | 210 | KEGG_WNT_SIGNALING_PATHWAY | 4.95E-05 |
| TIPIN | 179 | GO_ANTIGEN_PROCESSING_AND_PRESENTATION_VIA_MHC_CLASS_IB | 6.61E-04 |
| TMOD3 | 158 | GO_REGULATION_OF_RNA_SPLICING | 5.72E-05 |
| GINS1 | 151 | GO_DNA_REPLICATION | 2.67E-20 |
| PPM1F | 150 | GO_MEMBRANE_REGION | 1.05E-05 |
| DUT | 143 | GO_L_ASCORBIC_ACID_BINDING | 4.05E-04 |
| CDC20 | 141 | GO_PEPTIDYL_TYROSINE_MODIFICATION | 4.74E-04 |
| TPP1 | 140 | GO_VACUOLAR_LUMEN | 1.76E-06 |
| UCHL5 | 139 | GO_RESPONSE_TO_TOPOLOGICALLY_INCORRECT_PROTEIN | 7.36E-04 |
| TAF9B | 131 | GO_TRANSCRIPTION_FACTOR_COMPLEX | 5.06E-05 |
| SERPINE1 | 127 | GO_REGULATION_OF_N_METHYL_D_ASPARTATE_SELECTIVE_GLUTAMATE_ RECEPTOR_ACTIVITY | 6.12E-05 |
| KDR | 125 | GO_MEMBRANE_REGION | 6.99E-07 |
| SGSM2 | 122 | REACTOME_UNFOLDED_PROTEIN_RESPONSE | 3.92E-04 |
| MRTO4 | 119 | GO_RIBOSOME_BIOGENESIS | 6.91E-07 |
| SFRS1 | 112 | GO_POSITIVE_REGULATION_OF_GENE_EXPRESSION | 2.01E-04 |
| PGF | 111 | GO_TRIGLYCERIDE_CATABOLIC_PROCESS | 1.59E-04 |
| NISCH | 110 | REACTOME_UNFOLDED_PROTEIN_RESPONSE | 2.06E-04 |
| RBBP4 | 109 | GO_EXECUTION_PHASE_OF_APOPTOSIS | 2.14E-04 |
| TSPAN7 | 100 | GO_IMPORT_INTO_CELL | 2.51E-04 |

Key driver subnetworks in control Bayesian network with most top ranked key driver (KD) of subnetwork, number of nodes (size), most significantly overrepresented functional category and *P*-value.

**Supplementary Table 5 Extended list of key driver subnetworks in oxPAPC Bayesian network (Fig. 2b).**

| KD | size | Top functional canonical gene set category | p–value |
|---|---|---|---|
| CCNB2 | 513 | HALLMARK_E2F_TARGETS | 1.52E-20 |
| UBXN4 | 318 | GO_GOLGI_ORGANIZATION | 2.85E-05 |
| KIF4A | 304 | GO_CELL_CYCLE_PROCESS | 3.52E-14 |
| NDC80 | 263 | HALLMARK_E2F_TARGETS | 2.72E-14 |
| PCDH12 | 261 | GO_ANGIOGENESIS | 4.85E-05 |
| ASPM | 242 | GO_ORGANELLE_FISSION | 1.96E-09 |
| CCNB1 | 235 | GO_ANION_TRANSMEMBRANE_TRANSPORTING_ATPASE_ACTIVITY | 3.76E-04 |
| KDR | 214 | HALLMARK_ESTROGEN_RESPONSE_LATE | 1.96E-04 |
| CDCA3 | 184 | KEGG_ABC_TRANSPORTERS | 1.12E-05 |
| NUSAP1 | 175 | GO_DNA_REPLICATION | 2.71E-14 |
| PLK1 | 160 | GO_ANION_TRANSMEMBRANE_TRANSPORTING_ATPASE_ACTIVITY | 1.18E-04 |
| CDKN3 | 159 | GO_ANION_TRANSMEMBRANE_TRANSPORTING_ATPASE_ACTIVITY | 1.16E-04 |
| DPYSL2 | 155 | GO_IRON_ION_HOMEOSTASIS | 6.65E-04 |
| LOC100292189 | 139 | GO_CIRCULATORY_SYSTEM_DEVELOPMENT | 2.22E-04 |
| GRN | 138 | KEGG_LYSOSOME | 8.22E-11 |
| KIAA1462 | 136 | GO_REGULATION_OF_PHOSPHORUS_METABOLIC_PROCESS | 3.95E-06 |
| TMED2 | 131 | GO_ACTIVATION_OF_NF_KAPPAB_INDUCING_KINASE_ACTIVITY | 6.07E-04 |
| DNAJB1 | 129 | GO_PROTEIN_FOLDING | 6.55E-07 |
| EMG1 | 129 | GO_NCRNA_PROCESSING | 3.68E-05 |
| ASNS | 121 | GO_CELLULAR_AMINO_ACID_METABOLIC_PROCESS | 2.38E-09 |
| MTHFD2 | 114 | GO_CELLULAR_AMINO_ACID_METABOLIC_PROCESS | 7.32E-08 |
| PNMA2 | 114 | GO_CIRCULATORY_SYSTEM_DEVELOPMENT | 5.13E-04 |
| YWHAZ | 113 | BIOCARTA_ARAP_PATHWAY | 3.92E-04 |
| HSPA1A | 111 | GO_PROTEIN_FOLDING | 6.34E-06 |
| ARMCX2 | 109 | GO_CIRCULATORY_SYSTEM_DEVELOPMENT | 2.71E-04 |
| CHAC1 | 107 | REACTOME_CYTOSOLIC_TRNA_AMINOACYLATION | 2.98E-08 |
| SYPL1 | 102 | GO_NEGATIVE_REGULATION_OF_ERBB_SIGNALING_PATHWAY | 5.86E-05 |

Key driver subnetworks in oxPAPC Bayesian networks with most top ranked key driver (KD) of subnetwork, number of nodes (size), most significantly overrepresented functional category and *P*-value.

**Supplementary Table 6 Canonical pathway enrichment in MTHFD2 network.**

| MTHFD2 network: Top functional canonical gene set categories | p–value |
|---|---|
| **MTOR_UP.N4.V1_UP** | 5.35E-13 |
| **ALK_DN.V1_UP** | 2.23E-11 |
| **GO_CELLULAR_AMINO_ACID_METABOLIC_PROCESS** | 7.32E-08 |
| **KEGG_GLYCINE_SERINE_AND_THREONINE_METABOLISM** | 2.53E-06 |
| **REACTOME_CYTOSOLIC_TRNA_AMINOACYLATION** | 2.53E-06 |
| **GO_SERINE_FAMILY_AMINO_ACID_METABOLIC_PROCESS** | 2.53E-06 |
| **GO_TRNA_BINDING** | 2.53E-06 |
| **HALLMARK_MTORC1_SIGNALING** | 3.91E-06 |
| **GO_INTRINSIC_APOPTOTIC_SIGNALING_PATHWAY_IN_RESPONSE_TO_ENDOPLASMIC_RETICULUM_STRESS** | 6.39E-06 |
| **GO_ORGANIC_ACID_METABOLIC_PROCESS** | 7.46E-06 |
| **KEGG_AMINOACYL_TRNA_BIOSYNTHESIS** | 1.08E-05 |
| **REACTOME_TRNA_AMINOACYLATION** | 1.08E-05 |

The top 12 canonical gene sets (p-value) are shown. *MTOR* = mechanistic Target of Rapamycin, *ALK* = Anaplastic Lymphoma Kinase, *MTORC1* = MTOR complex 1.

**Supplementary Table 7: Clinical and demographic data of the human atherosclerosis cohort.**

| Characteristics | Healthy | Patients |
|---|---|---|
| **Demographic data** | | |
| No | 20 | 52 |
| Mean age (range) | 65 (50–82) | 68.7 (52–80) |
| Male /female | 10/10 | 32/20 |
| Smokers | 0 | 4 |
| **Clinical data** | | |
| Hypertension | 0 | 32 |
| Diabetes | 0 | 0 |
| Hyperlipidemia | 0 | 50 |
| Coronary disease | 0 | 12 |
| Myocardial Infraction | 0 | 0 |
| Valve insufficiency | 0 | 0 |
| Renal disease | 0 | 0 |
| Heart failure | 0 | 0 |
| **Angiographic carotid stenosis** | | |
| <90% | 0 | 52 |
| **Plaque histopathology** | | |
| Unstable | 0 | 26 |
| Stable | 0 | 26 |
| **Medication** | | |
| ACE inhibitors | 0 | 16 |
| β-blockers | 0 | 14 |

**Supplementary Table 8: Characteristics of the HDL study population.**

| | Healthy (n=10) | CAD (n=10) | p-value |
|---|---|---|---|
| Number of male subjects | 10 | 10 | |
| Age | 55 ± 10 | 62 ± 7 | 0.1384 |
| BP systolic (mmHg) | 124 ± 10 | 131 ± 18 | 0.3330 |
| BP diastolic (mmHg) | 83 ± 6 | 80 ± 11 | 0.5775 |
| Heart rate (bpm) | 57 ± 7 | 68 ± 15 | 0.0599 |
| LVEF (%) | 60 ± 5 | 57 ± 7 | 0.2785 |
| Glucose (mmol/l) | 5 ± 0.6 | 6.7 ± 1.5 | 0.0406 |
| HbA1c (%) | 5.4 ± 0.1 | 6.2 ± 1.1 | 0.0051 |
| Total cholesterol (mmol/l) | 5.2 ± 0.8 | 4.2 ± 1.4 | 0.0607 |
| HDL cholesterol (mmol/l) | 1.5 ± 0.4 | 1.1 ± 0.4 | 0.0235 |
| LDL cholesterol (mmol/l) | 3.2 ± 0.7 | 2.5 ± 1.1 | 0.0903 |
| Triglycerides (mmol/l) | 1.0 ± 0.2 | 1.8 ± 1.4 | 0.0864 |
| Creatinine (µmol/l) | 83.5 ± 12.1 | 82.7 ± 29.8 | 0.9383 |
| NT-proBNP (ng/l) | 46.8 ± 32.9 | 408.4 ± 524.7 | 0.0432 |
| Leukocytes (106/microl) | 5 ± 0.8 | 6.7 ± 1.3 | 0.0031 |
| Erythrocytes (106/microl) | 4.9 ± 0.3 | 4.7 ± 0.3 | 0.2811 |
| Hemoglobin (g/dl) | 145.2± 5.3 | 141.1 ± 10 | 0.2685 |
| Thrombocytes ($10^6$/µl) | 223.5 ± 90.4 | 213.9 ± 44.7 | 0.7668 |
| Hypertension | 0 | 9 | |
| Diabetes | 0 | 2 | |
| Obesity | 0 | 7 | |
| Dyslipidemia | 0 | 10 | |
| MI History | 1 | 2 | |

BMI = body mass index, LVEF = left ventricular ejection fraction, BP = blood pressure, NT-proBNP = N-terminal pro brain natriuretic peptide, CK = creatine kinase, MI = myocardial infarction. Data are represented as mean ± SD (Student's t-test).

**Supplementary Table 9: List of primers for RT-qPCR.**

## List of Human Primers

| Name | Forward Primer (5'-3') | Reverse Primer (5'-3') |
|---|---|---|
| ß-Actin | AAAGACCTGTACGCCAACAC | GTCATACTCCTGCTTGCTGAT |
| MTHFD2 | GATCCTGGTTGGCGAGAATCC | TCTGGAAGAGGCAACTGAACA |
| SHMT2 | CCCTTCTGCAACCTCACGAC | TGAGCTTATAGGGCATAGACTCG |
| PHGDH | CTGCGGAAAGTGCTCATCAGT | TGGCAGAGCGAACAATAAGGC |
| PSAT1 | TGCCGCACTCAGTGTTGTTAG | GCAATTCCCGCACAAGATTCT |
| MTHFD1L | CTGCCTTCAAGCCGGTTCTT | TTTCCTGCATCAAGTTGTCGT |
| GARS | ATGGAGGTGTTAGTGGTCTGT | CTGTTCCTCTTGGATAAAGTGCT |
| CARS | GGTGACGTGGTATTGCTGTG | CTCTTCTCCCGATACTGCTCG |
| CEBPB | ACAAGCACAGCGACGAGTACAAGA | TGCTTGAACAAGTTCCGCAGGGT |
| SLC7A5 | CCGTGAACTGCTACAGCGT | CTTCCCGATCTGGACGAAGC |
| SLC7A1 | GTCCTGCTCAACATTGGGCA | CAGGGCCTGCATTCTCACG |
| ASNS | GGAAGACAGCCCCGATTTACT | AGCACGAACTGTTGTAATGTCA |
| DDIT3 | AGCTGGAAGCCTGGTATGAG | AGTCAGCCAAGCCAGAGAAG |
| ATF4 | CTGCCCGTCCCAAACCTTAC | TGCTCCGCCCTCTTCTTCTG |
| PCK2 | GCCATCATGCCGTAGCATC | AGCCTCAGTTCCATCACAGAT |
| PFKFB3 | GGGCCAAAGCTGACCAACTC | CCCTTCTTTCGCCAGGTAGC |
| G6PD | CGAGGCCGTCACCAAGAAC | GTAGTGGTCGATGCGGTAGA |
| EHHADH | AAACTCAGACCCGGTTGAAGA | TTGCAGAGTCTACGGGATTCT |
| CTH | GGCCTGGTGTCTGTTAATTGT | GCCATTCCGTTTTTGAAATGCT |
| CBS | GGCCAAGTGTGAGTTCTTCAA | GGCTCGATAATCGTGTCCCC |
| F3 | GGAACCCAAACCCGTCAATC | GCCAAGTACGTCTGCTTCAC |
| KDM7A | ACCTGAATGGAGAGCGAAAG | TCATGTTCCACTCCCTCTAC |
| GSS | GGGAGCCTCTTGCAGGATAAA | GAATGGGGCATAGCTCACCAC |
| sXBP1 | CCGCAGCAGGTGCAGG | GAGTCAATACCGCCAGAATCCA |
| GCLM | CATTTACAGCCTTACTGGGAGG | ATGCAGTCAAATCTGGTGGCA |
| 18srRNA | CTTTGGTCGCTCGCTCCTC | CTGACCGGGTTGGTTTTGAT |

## List of Mouse Primers

| Name | Forward Primer (5'-3') | Reverse Primer (5'-3') |
|---|---|---|
| Mthfd2 | GCCCAAATTGGTTGGAGATG | CGCTGTTTGGACTTGAACAC |
| Phgdh | ATGGCCTTCGCAAATCTGC | AGTTCAGCTATCAGCTCCTCC |
| Shmt2 | TGGCAAGAGATACTACGGAGG | GCAGGTCCAACCCCATGAT |
| Slc3a2 | TGATGAATGCACCCTTGTACTTG | GCTCCCCAGTGAAAGTGGA |

## List of Zebrafish Primers

| Name | Forward Primer (5’-3’) | Reverse Primer (5’-3’) |
|---|---|---|
| elfa | CTTCTCAGGCTGACTGTGC | CCGCTAGCATTACCCTCC |
| mthfd2 | CGGGCATTGCGGAAACTCTG | CTGGCTGGATTGTCACCTAC |
