# Supplementary material for: Oxidized phospholipids regulate amino acid metabolism through MTHFD2 to facilitate nucleotide release in endothelial cells
Source: Nat Commun. 2018 Jun 12;9:2292. doi: 10.1038/s41467-018-04602-0 (PMC5997752; doi:10.1038/s41467-018-04602-0)
Supplement: Supplementary file 2 — Description of Additional Supplementary Files [file 41467_2018_4602_MOESM2_ESM.pdf]

Description of Additional Supplementary Files

File Name: Supplementary Data 1
Description: Edge table of control Bayesian network

File Name: Supplementary Data 2
Description: Edge table of oxPAPC Bayesian network

File Name: Supplementary Data 3
Description: Gene set enrichment analysis of MTHFD2 RNAseq signature. Enrichment of differentially expressed genes (Fig 4a, FDR < 0.05) in canonical and non-canonical gene set categories (MSigDB v6.0) (FET P-value < 1E-04). Gene set category, number of genes in gene set category, number of genes which overlap with RNAseq signature, odds ratio, P -value and overlapping genes are listed.

File Name: Supplementary Data 4
Description: Association between genes of the MTHFD2 network and plasma metabolites. List of genes in MTHFD2 network with SNPs (P-value < 1E-04) associated with metabolites in human blood with physical locations of the variant within ±5 kb of gene bodies.

File Name: Supplementary Data 5
Description: Genetic variances in genes of the MTHFD2 network associated with CAD risk. List of genes within the MTHFD2 network with SNPs (P-value < 1E-04) associated with Coronary artery disease (CAD) and myocardial infarction based on the CARDIoGRAMplusC4D consortium with physical locations of the variant within ±500 kb of gene bodies
